# Homologous recombination DNA repair deficiency and PARP inhibition activity in primary triple negative breast cancer

**DOI:** 10.1038/s41467-020-16142-7

**Published:** 2020-05-29

**Authors:** Neha Chopra, Holly Tovey, Alex Pearson, Ros Cutts, Christy Toms, Paula Proszek, Michael Hubank, Mitch Dowsett, Andrew Dodson, Frances Daley, Divya Kriplani, Heidi Gevensleben, Helen Ruth Davies, Andrea Degasperi, Rebecca Roylance, Stephen Chan, Andrew Tutt, Anthony Skene, Abigail Evans, Judith M. Bliss, Serena Nik-Zainal, Nicholas C. Turner

**Affiliations:** 1 Breast Cancer Now Toby Robins Research Centre, The Institute of Cancer Research, London, CB2 0XZ United Kingdom; 2 Clinical Trials and Statistics Unit, The Institute of Cancer Research, London, United Kingdom; 3 The Centre for Molecular Pathology, The Royal Marsden Hospital, 15 Cotswold Road, Sutton, SM2 5NG Surrey United Kingdom; 4 Ralph Lauren Centre for Breast Cancer Research, Royal Marsden Hospital, London, United Kingdom; 5 Department of Medical Genetics, The Clinical School, Box 238, Level 6 Addenbrooke’s Treatment Centre, Cambridge Biomedical Campus, Cambridge, CB2 0QQ United Kingdom; 6 MRC Cancer Unit, Hutchison/MRC Research Centre, University of Cambridge, Box 197, Cambridge Biomedical Campus, Cambridge, CB2 0XZ United Kingdom; 7 University College London Hospitals NHS Foundation Trust, NIHR University College London Hospitals Biomedical Research Centre, London, United Kingdom; 8 Nottingham University Hospital Trust (City Campus), Nottingham, United Kingdom; 9 Breast Cancer Now Research Unit, Cancer Centre, Guy’s Hospital, King’s College London, London, United Kingdom; 10 Royal Bournemouth Hospital, Bournemouth, United Kingdom; 11 Poole Hospital NHS Foundation Trust, Poole, United Kingdom; 12 Breast Unit, The Royal Marsden Hospital, Fulham Road, London, United Kingdom

**Keywords:** Cancer screening, Breast cancer

## Abstract

Triple negative breast cancer (TNBC) encompasses molecularly different subgroups, with a subgroup harboring evidence of defective homologous recombination (HR) DNA repair. Here, within a phase 2 window clinical trial, RIO trial (EudraCT 2014-003319-12), we investigate the activity of PARP inhibitors in 43 patients with untreated TNBC. The primary end point, decreased Ki67, occured in 12% of TNBC. In secondary end point analyses, HR deficiency was identified in 69% of TNBC with the mutational-signature-based HRDetect assay. Cancers with HRDetect mutational signatures of HR deficiency had a functional defect in HR, assessed by impaired RAD51 foci formation on end of treatment biopsy. Following rucaparib treatment there was no association of Ki67 change with HR deficiency. In contrast, early circulating tumor DNA dynamics identified activity of rucaparib, with end of treatment ctDNA levels suppressed by rucaparib in mutation-signature HR-deficient cancers. In ad hoc analysis, rucaparib induced expression of interferon response genes in HR-deficient cancers. The majority of TNBCs have a defect in DNA repair, identifiable by mutational signature analysis, that may be targetable with PARP inhibitors.

## Abstract (web summary)

Defects in homologous recombination (HR) are found in some triple negative breast cancers, suggesting they may be sensitive to PARP inhibitors. In this phase II clinical trial of the PARP inhibitor rucaparib, changes in Ki67 levels did not correlate with markers of HR deficiency but HR deficiency was detected in 69% of tumours, indicating that PARP inhibitors may be a useful treatment.

## Introduction

Triple negative breast cancer (TNBC) may have diverse defects in HR DNA repair, through germline mutations in *BRCA1*, *BRCA2* and *PALB2*, somatic mutations in *BRCA1* and *BRCA2*, promoter methylation of *BRCA1* and *RAD51C*, and other as yet to be identified mechanisms^1–4^. Over the last decade, advances in whole-genome sequencing (WGS) have lead to the identification of mutational processes that leave a characteristic imprint, a mutational signature on the cancer genome. These have revolutionalised our understanding of cancer and has the capability to improve diagnosis and treatment of cancer^5,6^.

Cancers with defects in HR-based DNA repair have characteristic chromosomal changes reflecting the use of alternative error-prone repair pathways, including measures of genomic instability; loss of heterozygosity, telomeric allelic imbalance and large-scale state transitions to accurately identify *BRCA1/2* tumours^7–9^, and their combination to form the HRD Score has allowed identification of HR-deficient tumours (HRD Score >42), independent of *BRCA1/2* deficiency within a sporadic TNBC population^10^. Recent work has identified WGS signatures of HR deficiency with *BRCA1/2* deficient tumours associated with distinct mutational signatures. The mutational signatures and chromosomal instability markers of HR deficiency have been aggregated into the HRDetect score, robustly identifying *BRCA1/2* tumours with potential greater accuracy than indexes such as HRD-score^11,12^.

Whether mutational signature-based scores such as HRDetect, can be used to direct therapy in the clinic is unknown, in part as there is limited direct evidence that cancers classified as HR deficient by these scores have a functional defect in HR. Breast cancers with *BRCA1* and *BRCA2* germline mutations are highly sensitive to PARP inhibitors^13,14^, which target the underlying HR DNA repair defect in these cancers. However, no activity was observed with PARP inhibitors in the treatment of heavily pre-treated un-selected advanced TNBC^15^. The extent to which this PARP inhibitor efficacy may translate to sporadic TNBC is unknown, as is the best way to identify HR-deficient TNBC. To address these questions, we designed a translational clinical trial, the RIO trial (EudraCT 2014-003319-12), with the objective of identifying biomarkers of PARP inhibitor activity in sporadic TNBC.

## Results

### Biomarkers of HR deficiency in primary TNBC

**Fig. 1:**
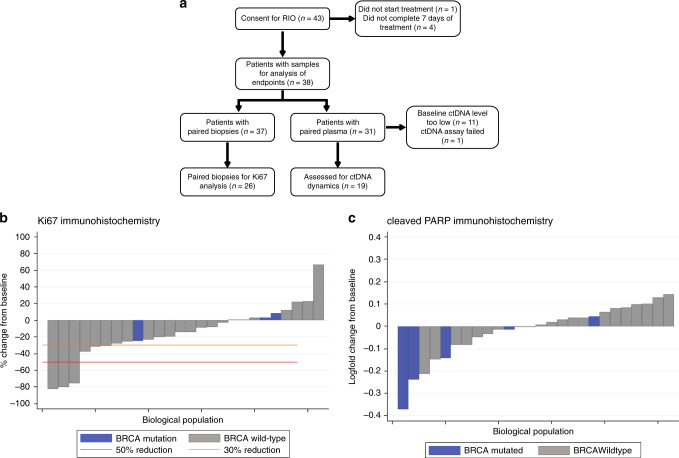
RIO study CONSORT diagram and HRDetect analysis. **a** RIO study CONSORT diagram. **b** Effect of rucaparib on Ki67 expression assessed by immunohistochemistry (IHC). The change in proportion of tumor cells expressing Ki67 between baseline and EOT, in patients that had assessable pairs of baseline and EOT samples. *BRCA* mutation cancers had no evidence of decreased Ki67. **c** Effect of rucaparib on cleaved PARP expression assessed by immunohistochemistry, as a marker of apoptosis. The change in proportion of tumor cells expressing cleaved PARP between baseline and EOT, in patients that had assessable pairs of baseline and EOT samples. *BRCA* mutation cancers had no evidence of increased cleaved PARP expression. Grey bars, *BRCA* wild type patients; Blue bars, *BRCA* germline mutant patients. Orange line, >30% but <50% reduction; Red line, >50% reduction.

Patients with newly diagnosed, treatment naïve TNBC were treated with the PARP inhibitor rucaparib for 2 weeks prior to surgery or neoadjuvant chemotherapy. A total of 43 patients were entered into the trial between August 2015 and August 2017. Blood and tissue biopsies were taken prior to, and at the end of treatment, for molecular analysis (Fig. 1a). Within the trial, a subset of germline *BRCA1/2* patients were recruited as a control population. The trial prospectively examined three potential biomarkers of PARP inhibitor activity, a molecular signature of HR deficiency using HRDetect, RAD51 focus formation in a tumor biopsy at the end of treatment, and *BRCA1* methylation. The primary activity end point was a fall in Ki67 on the end of treatment biopsy, with circulating tumor DNA dynamics as a prospectively planned exploratory end point of activity. Patient demographics were as expected for this population (Table 1). Rucaparib was well tolerated with adverse effect profile similar to previous clinical studies^16,17^ (Supplementary Table 1).

**Table 1:** RIO study patient demographics.

		*n*	%
*Age (mean (standard deviation))*		54.6 (13.9)	
Age group (years)	<40	7	16.3
	40-49	13	30.2
	50-59	9	20.9
	60-69	5	11.6
	70+	9	20.9
BRCA status	Triple negative, known *BRCA*1/2 carrier at registration	2	4.7
	Not TN, known *BRCA1/2* mutation carrier at registration	1	2.3
	Triple neg, no BRCA mutation	35	81.4
	Triple negative, BRCA1/2 mutation identified while on trial	5	11.6
Planned standard treatment after rucaparib	Neoadjuvant chemotherapy	32	74.4
	Surgical resection	11	25.6
Hormone receptor status	ER & PR negative^a^	42	97.7
	ER positive & PR negative	1	2.3
Tumour grade (diagnostic sample)	G1	0	0
	G2	12	27.9
	G3	24	55.8
	Not known	7	16.3
Histological type	Infiltrating ductal	38	88.4
	Infiltrating lobular	4	9.3
	Mixed ductal & lobular	1	2.3
DCIS present	Yes	7	16.3
	No	35	81.4
	Not known	1	2.3
Tumour size by ultrasound	<1.5	2	4.7
	1.5	1	2.3
	>1.5 & ≤2	10	23.3
	>2 & ≤5	26	60.5
	>5	4	9.3
Lymph node involvement	Yes	16	37.2
	No	27	62.8
Side of tumour	Left	18	41.9
	Right	25	58.1
Evidence of metastatic disease	Yes	0	0
	No	43	100

^a^One patient was locally assessed as triple negative but central assessment noted weak PgR score of 3/8 by Allred.

We assessed rucaparib activity and the relationship with prospectively planned biomarkers. The primary end point was assessed in tissue samples, using Ki67 suppression after two weeks assessed by immunohistochemistry, as a potential biomarker (Fig. 1b). A drop in Ki67 by 50% in the triple negative patients without a known *BRCA1/2* mutation at trial entry was seen in 12% tumors (95% CI: 2.5–31.2; *n* = 3/25). In secondary end point analysis, one additional patient with known BRCA mutation at trial entry was assessed and did not have a 50% drop in Ki67 (Fig. 1b). No association was observed between Ki67 change with *BRCA1/2* mutated cancers (Fig. 1b). Similarly, no association was observed with cleaved PARP levels as a marker of apoptosis with *BRCA1/2* mutated cancers (Fig. 1c).

**Fig. 2:**
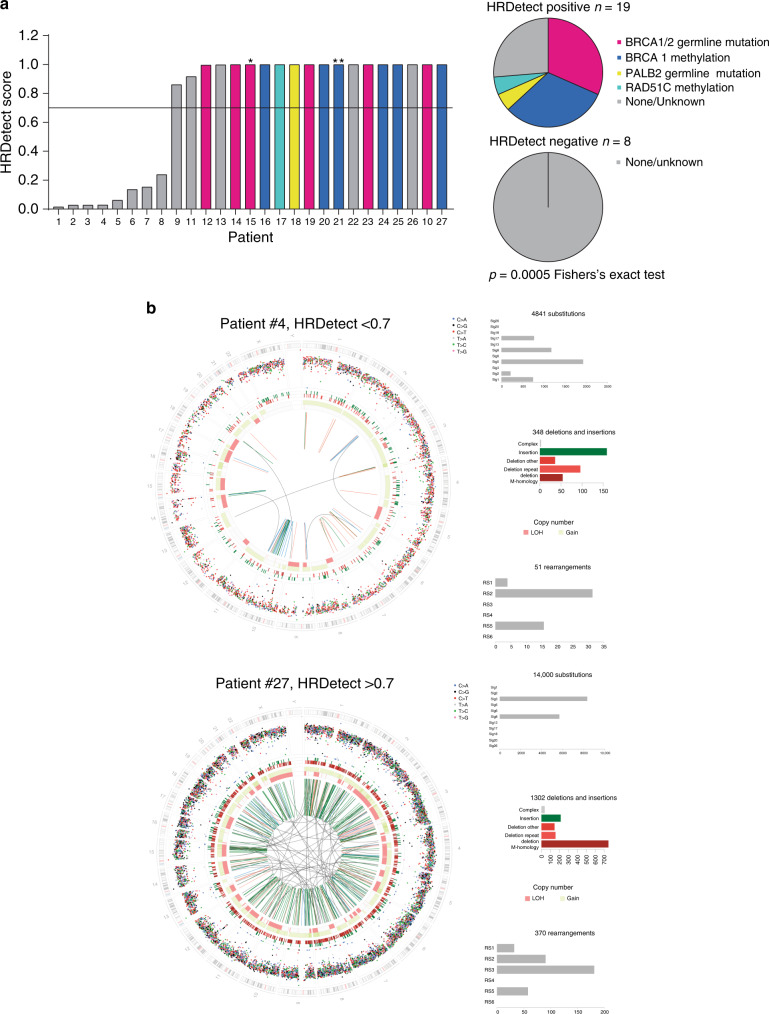
HRDetect analysis. **a** HRDetect scores were established with whole-genome sequencing in baseline biopsies of 26 patients with untreated primary triple negative breast cancer (locally assessed) entering the RIO window clinical trial. HRDetect positive cancers (HRDetect score >0.7) were enriched for inactivating mutations and promoter methylation of HR genes compared to HRDetect negative cancers (*p* = 0.0005, Fisher’s exact test). Star, an additional patient with ER-positive breast cancer and *BRCA2* germline mutation is shown. 2 stars, locally assessed as TNBC but centrally assessed as PR-positive breast cancer with *BRCA1* methylation. Magenta bars, *BRCA1/2* germline mutation; Blue bars, *BRCA1* methylation; Yellow bars, *PALB2* germline mutation; Turquoise bars, *RAD51C* methylation; Grey bar, None/unknown. **b** Examples of genome plots for a sample with low (top) and high (bottom) HRDetect scores. The histograms associated with each circos plot show mutation counts for each mutation class: the topmost histogram shows the number of mutations contributing to each substitution signature; the middle histogram represents indel patterns; and the bottom histogram shows the number of rearrangements contributing to each rearrangement signature.

In secondary end point analysis, baseline biopsies of patients entering the trial, we interrogated the prevalence of HR deficiency in primary TNBC (Fig. 2 and Supplementary Table 2). We performed whole-genome sequencing analysed with the HRDetect assay^11^, identifying mutational processes characteristic of HR deficiency in 69% locally assessed TNBC (18/26 with score >0.70, herewith called HRDetect+ve; Fig. 2a,b and Supplementary Fig. 1a) as well as an additional control ER-positive cancer with a known germline *BRCA2* mutation. In ad hoc analysis, we individually determined the mutational status of *BRCA1, BRCA2* and *PALB2*, and promoter methylation of *BRCA1* and *RAD51C* (Fig. 2a). Of the HRDetect+ve cancers, 74%(14/19) had a detectable underlying mutation of *BRCA1/2* and *PALB2* or gene promoter hypermethylation of *BRCA1* or *RAD51C*. None of the eight HRDetect−ve cancers had an underlying genetic/epigenetic defect (*p* = 0.0005, Fisher’s exact test; Fig. 2a). A loss of heterozygosity (LOH)/copy-number-based HRD score was positive in more cancers than HRDetect, with the HRD score identifying cancers with genomic instability but without rearrangement signatures and indels at microhomology. None of the HRD score high but HRDetect low tumours had detectable pathway aberrations (Supplementary Fig. 1b), suggesting that HRDetect was more specific.

**Fig. 3:**
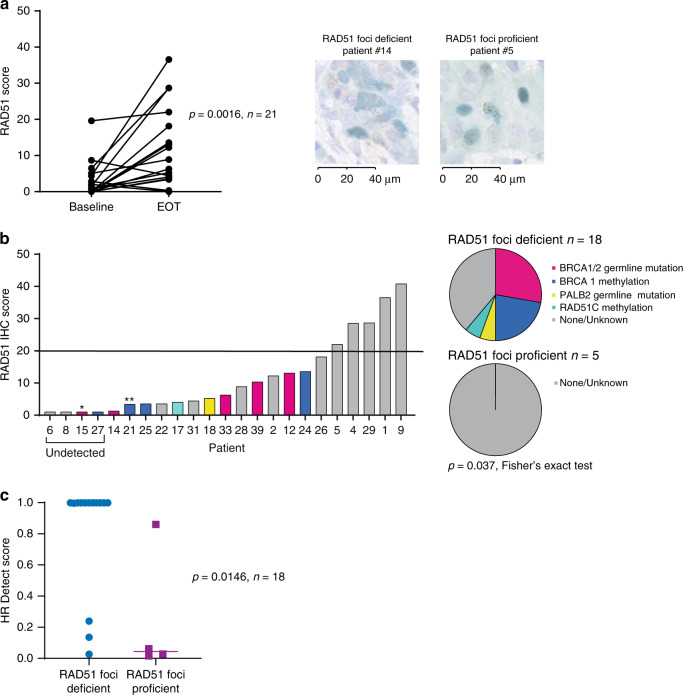
Biomarkers of homologous recombination (HR) repair deficiency in TNBC. **a** RAD51 focus assessment in paired baseline and on-treatment biopsies, *p* = 0.0016 Wilcoxon test. Inset right, example immunohistochemistry images of two cancers, one deficient and one proficient in RAD51 focus formation (brown), in nuclei stained for geminin (blue). Scale bars show 40 μm. **b** RAD51 immunohistochemistry score (>20% RAD51 foci proficient), as a functional assessment of HR proficiency, was assessed in end of treatment biopsies after 2 weeks of rucaparib in 25 patients. RAD51 foci deficient cancers are enriched for inactivating mutations and promoter methylation of HR genes compared to RAD51 proficient cancers (*p* = 0.037 Fisher’s exact test). *ER-positive breast cancer and *BRCA2* germline mutation is shown. **Locally assessed TNBC but centrally assessed as PR-positive breast cancer with *BRCA1* methylation. Magenta bars, *BRCA1/2* germline mutation; Blue bars, *BRCA1* methylation; Yellow bars, *PALB2* germline mutation; Turquoise bars, *RAD51C* methylation; Grey bars, None/unknown. **c** Association between RAD51 foci proficiency and HRDetect scores in 18 patients, *p* = 0.0146 Mann–Whitney *U* Test. Line indicates median level.

HRDetect identified all cancers with known HR pathway defects, as well as additional sporadic cancers with no single detectable defect (Fig. 2a). In secondary end point analysis, we next addressed whether HRDetect+ve cancers had an underlying functional defect in HR DNA repair, using RAD51 focus formation in the end of treatment (EOT) biopsy. When cells are exposed to genotoxic agents such as PARP inhibition, RAD51 is recruited to sites of DNA damage and stalled replication forks, mediating the search for a homologous sequence during HR^18^, with RAD51 nuclear foci visible at sites of repair as a hallmark for HR-mediated repair^19^. The impaired ability to form RAD51 foci after DNA damage may identify cancers with defective HR^20^. We developed a novel immunohistochemistry assay to assess RAD51 foci, co-staining with geminin (GMNN) to identify cells in S/G2 phase of the cell cycle and after cytotoxic treatment; RAD51 score <20% (less than 20% geminin positive cells having RAD51 foci, RAD51 foci deficiency) was assessed to indicate HR deficiency in an independent sample set (Supplementary Fig. 2). Within the RIO trial, RAD51 IHC scores increased significantly from baseline to EOT (*p* = 0.0016, Wilcoxon test), reflecting rucaparib induced DNA damage and RAD51-mediated repair (Fig. 3a). In EOT biopsies, RAD51 foci deficiency was identified in 77% (17/22) locally assessed TNBC, as well as an ER-positive *BRCA2* mutant control cancer (Fig. 3b). Of the RAD51-deficient cancers, 61% (11/18) had an underlying detectable HR defect compared to none (0/5) of RAD51 foci proficient cancers (*p* = 0.037 Fisher’s exact test; Fig. 3b). Cancers with RAD51 foci deficiency had significantly higher HRDetect scores than tumour samples that were RAD51 foci proficient (*n* = 18, *p* = 0.0146 Mann–Whitney test; Fig. 3c). HRDetect therefore identified cancers with a functional defect in HR-based DNA repair, with functional HR deficiency occurring in the majority of TNBC.

### Rucaparib activity assessed by ctDNA dynamics

**Fig. 4:**
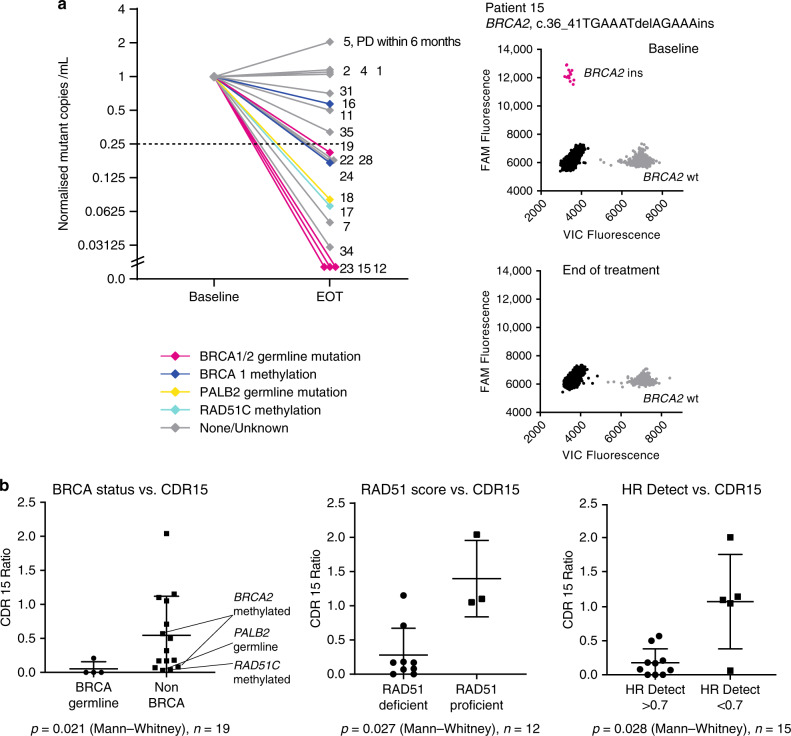
ctDNA dynamics reveals activity of rucaparib in primary triple negative homologous recombination repair deficient cancers. **a** Change in circulating tumour DNA (ctDNA) copies/ml between baseline and end of treatment (EOT) after two weeks of rucaparib. The relative change of on-treatment ctDNA levels (Circulating tumor DNA ratio, CDR15) with HR pathway defects indicated. Right, example digital PCR ctDNA analysis plots. Magenta lines, *BRCA1/2* germline mutation; Blue lines, *BRCA1* methylation; Yellow lines, *PALB2* germline mutation; Turquoise lines, *RAD51C* methylation; Grey lines, None/unknown. **b** Associations of ctDNA change on rucaparib at day 15 (CDR15) with left, *BRCA1/2* germline mutations (*n* = 19 patients), middle, RAD51 focus proficiency (*n* = 12 patients) and right, HRDetect score (*n* = 15 patients). Centre line, mean; error bars, standard deviation. *p* values Mann–Whitney *U* test.

Circulating tumour DNA (ctDNA) is released from the tumor, allowing for serial sampling through the course of treatment^21,22^. Early changes in ctDNA dynamics represent an early biomarker of drug activity, as cancers that respond to treatment rapidly suppress the level of ctDNA in plasma^23–25^. Analysis of ctDNA was prospectively planned as an exploratory end point of rucaparib activity, in part, as Ki67 change has only been validated as an activity end point in endocrine based therapies^26–28^. To assess ctDNA in RIO, the primary tumor was sequenced in 35 patients, with somatic mutations identified in 31 patients, and personalised digital PCR used to track changes in ctDNA levels between baseline and end of treatment plasma (EOT). Change in ctDNA was assessable in the 19 patients with sufficiently high baseline ctDNA to assess change (Figs. 1a and 4a and Supplementary Tables 4 and 5). In contrast to the tumor biopsy-based data, a substantial proportion of patients suppressed ctDNA after rucaparib treatment (Fig. 4a).

Patients with germline *BRCA1/2* mutations did not suppress Ki67, nor induce PARP cleavage, in the end of treatment biopsy (Fig. 1). In contrast, patients with germline *BRCA1/2* mutations had a greater suppression of ctDNA than patients without germline mutations (*n* = 19, *p* = 0.021, Mann–Whitney; Fig. 4b), validating ctDNA dynamics as a marker of rucaparib activity. Cancers with deficient RAD51 foci formation (*n* = 12, *p* = 0.033, Mann–Whitney) and HRDetect+ve cancers had greater ctDNA suppression (*n* = 15, *p* = 0.027, Mann–Whitney; Fig. 4b). In ad hoc analysis patients with suppressed ctDNA after two weeks rucaparib (ctDNA ratio (CDR) <0.25, methods) were enriched for germline mutations of *BRCA1/2* and *PALB2* and gene promoter methylation of *BRCA1* and *RAD51C* (Fig. 4b). These data illustrate the potential of ctDNA analysis to transform window trials, presenting a simple and robust assay of drug activity, without the potential sampling challenges involved with repeat biopsies. However, analytical challenges associated with low plasma DNA levels and low purity tumor samples, will benefit from further technological development.

### PARP inhibition induces an interferon response in HR-deficient cancers

**Fig. 5:**
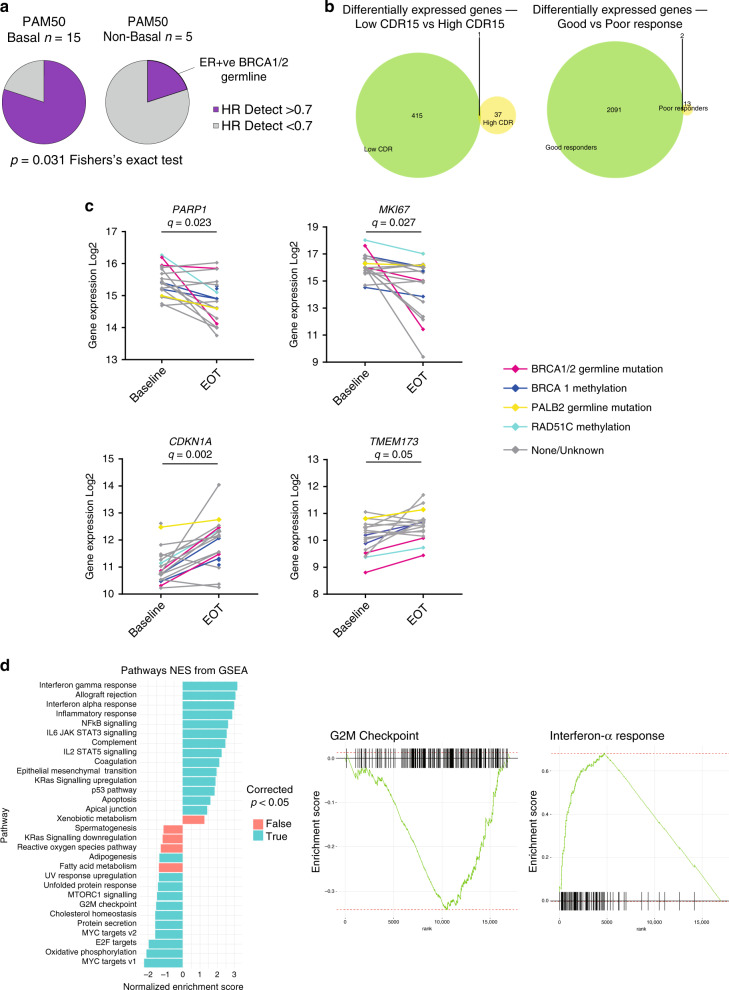
Expression analysis of primary triple negative homologous recombination repair deficient cancers. **a** Association between basal-like and non-basal-like triple negative subtypes, assessed by PAM50, and HRDetect score. *p* value Fisher’s exact test. *n* = 20 paired tumour samples. Purple, HRDetect score >0.7; Grey, HRDetect score <0.7. **b** Change in gene expression on paired tumor biopsies between baseline and end of treatment on rucaparib. Number of genes with a significant change in gene expression (Log fold change >0.5 and false discovery corrected *q* value < 0.1). Left, categorised by ctDNA suppression or not (CDR15 response <0.25 vs ≥1, *n* = 8 and *n* = 3 paired tumour samples respectively). Right, by CDR15 response and HRD score (CDR15 < 0.25 and HRD > 0.7 vs CDR15 ≥ 1 and HRD < 0.7, *n* = 11 and *n* = 6 paired tumour samples respectively). **c** Gene expression changes of *PARP1, MKI67, CDKN1A and TMEM173* through treatment, from DESeq2 with false discovery rate (FDR) corrected *q* value for change. *n* = 20 paired tumour samples. Magenta lines, *BRCA1/2* germline mutation; Blue lines, *BRCA1* methylation; Yellow lines, *PALB2* germline mutation; Turquoise lines, *RAD51C* methylation; Grey lines, None/unknown. **d** Left, Gene set enrichment pathway analysis (GSEA) for gene expression changes through treatment in patients with ctDNA suppression (CDR15 < 0.25, *n* = 8 paired tumour samples.) on rucaparib. Centre, suppression of G2M checkpoint genes on PARP inhibition, *q* = 0.006, and right, increased expression of interferon pathway genes on PARP inhibition, *q* = 0.001. False discovery rate corrected *q* value for change.

Having demonstrated that HRDetect identifies sporadic TNBC in which PARP inhibitors have activity, in ad hoc analysis we investigated the mechanisms of PARP inhibition in these cancers with RNA exome sequencing in 20 paired tumour samples, baseline and end of treatment. The majority of tumours were basal-like by PAM50 (75%, 15/20), with HRDetect scores >0.7 found in 80% (12/15) basal-like TNBC and none (0/4) of the non-basal TNBC, with one non-basal *BRCA1* mutant ER+ tumour having an HRDscore >0.7 (*p* = 0.031 Fisher’s exact test, Fig. 5a). Analysis of the immune micro-environment of baseline samples with CIBERSORT^29^ suggested higher levels of follicular helper T cells (*p* = 0.0126, *n* = 20 patients) in HRDetect+ve cancers along with higher levels of activated macrophages (Supplementary Fig. 3a and b).

Cancers with ctDNA suppression (CDR < 0.25) had more substantial changes in gene expression than cancers without ctDNA suppression (CDR > 0.25), with 415 compared to 37 significantly differentially expressed genes respectively (Fig. 5b), demonstrating stability of gene expression through rucaparib treatment in HR-proficient cancers. For individual genes, a significant decrease in gene expression was noted in *PARP1* mRNA (*q* = 0.023, *n* = 20, DESeq2 with false discovery rate (FDR) correction), *MKI67* mRNA (Ki67, *q* = 0.027, *n* = 20, DESeq2 FDR correction), as well as induction in gene expression of both *CDKN1A* mRNA (p21, *q* = 0.002, *n* = 20, DESeq2 FDR correction) and *TMEM173* mRNA (STING, *q* = 0.05, *n* = 20, DESeq2 FDR correction; Fig. 5c). In HRDetect+ve cancers (Supplementary Fig. 4) and cancers with associated ctDNA suppression (Fig. 5d) pathway analysis demonstrated significant gene expression changes in pathways that regulate proliferation, apoptosis and immune function. There was decreased expression of G2M checkpoint genes, reflecting cell cycle arrest with rucaparib (Fig. 5d). There was substantial increase in interferon response genes, which along with *TMEM173* mRNA expression, suggested PARP inhibition activated the cGAS–cGAMP–STING pathway in HR-deficient cancers (Fig. 5d and Supplementary Fig. 4). In contrast, HRDetect-ve cancers did not induce interferon response genes, nor had expression changes of cell cycle arrest, on rucaparib (Supplementary Fig. 4).

## Discussion

Results from the RIO trial demonstrate that a majority of primary TNBC have defects in HR-based DNA repair, reinforcing the companion population-based TNBC study by Staaf et al^4^. The primary end point of RIO, suppression of Ki67 in on-treatment biopsies, was infrequent. In pre-planned secondary analyses, we show that cancers with HR deficiency can be robustly identified with the mutational-signatures based classifier HRDetect (Fig. 2 and Supplementary Fig. 1), which identifies cancers with a functional deficiency in HR (Fig. 3b), and with evidence of activity of PARP inhibitors restricted to these cancers using ctDNA analysis (Fig. 3c). In ad hoc analysis, HRDetect was more specific to underlying HR deficiency than HRD scores (Supplementary Fig. 1b), suggesting that mutational signature assessment might be more accurate in identifying cancers that would benefit from platinum chemotherapy or PARP inhibition^4,30–32^.

Induction of RAD51 nuclear foci after neoadjuvant chemotherapy and PARP inhibition can measure the homologous recombination functionality in breast cancer biopsies^20,33–36^, with an association to loss of heterozygosity measures of HR deficiency^34,37^. Studies have shown that cells with deficient *BRCA1/2* or other HR proteins, do not efficiently form RAD51 foci which could be used as a marker for PARP inhibitor sensitivity using FFPE tumour samples^35,38,39^. The dynamics of DNA repair alter throughout tumour evolution and a functional RAD51 assay can be used as a dynamic readout of tumour HR status at the specific time for treatment decision-making^33,40,41^. Current studies are using immunofluorescence (IF) on FFPE samples which can be labour intensive, and here we develop a RAD51 foci immunohistochemistry (IHC). Our cutoff <20% for HR deficiency is consistent with the RECAP test which has recently shown to be effective in ascertaining HR deficiency in metastatic breast tumours treated with ionising radiation^36^. Further validation of this novel HR deficient biomarker is required and if clinically validated could be a useful tool in the clinic.

One of the main limitations of the study was lower recruitment into the trial than was anticipated. This was possibly due to patient preference to start treatment without the possibility of delaying for short-term trial therapy. Additionally, failure of tissue biopsies containing enough tumour content impacted end point analysis. We therefore demonstrate the advantage of non-invasive analysis, and the use of ctDNA as a potentially reliable and effective surrogate end point to assess response. This will require further validation.

We demonstrate that PARP inhibitors induce a pro-inflammatory/interferon response in HR-deficient TNBC, likely though the cGAS–cGAMP–STING pathway. Consistent with our findings, PARP inhibition has previously been shown to induce T-cell recruitment through activation of the cGAS-cGAMP-STING pathway in a *BRCA1*-null mouse model of TNBC^42^. Similarly, PARP inhibitors have been shown to upregulate interferon response in TNBC cell lines with BRCA2 depletion^43^ or mutant *BRCA1*^44^. Furthermore, Sceneay et al^45^ recently demonstrated using mouse models of TNBC, that immune dysfunction characterised by decreased interferon signalling and decreased antigen presentation was abrogated by a STING agonists. Together these findings underline the potential of exploiting immune dysfunction in the context of HR deficiency, notably in *BRCA1/2* mutant tumours, and TNBC more generally.

Our findings illustrate the potential of using whole-genome sequencing mutational signatures to guide cancer treatment, advocating for clinical trials of PARP inhibitors, potentially in combination with PD(L)1 targeting immune checkpoint antibodies, in sporadic TNBC with HR deficiency.

## Methods

### Study Design

Conducted in 10 hospitals throughout the United Kingdom, the window study of the PARP inhibitor rucaparib in patients with primary triple negative or BRCA1/2 related breast cancer (The RIO study; EudraCT 2014-003319-12, Cancer Research UK trial CRUK/12/034) was a single-group, open-label, phase II window of opportunity trial assessing rucaparib efficacy in patients with primary triple negative or *BRCA1/2* mutant breast cancer prior to commencing primary treatment (neoadjuvant chemotherapy or surgery). The trial was co-sponsored by the Institute of Cancer Research and the Royal Marsden Hospital NHS Foundation Trust. Ethical approval for The RIO trial was given by the NRES Committee London - Fulham Research Ethics Committee (REC ID: 14/LO/2181) and informed consent was obtained from all patients enrolled in the study.

Key eligibility criteria include breast tumour size ≥2 or <2 cm with cytologically/histologically confirmed axillary lymph nodes, WHO performance status 0–2, no prior history of ipsilateral breast cancer within 5 years and no prior treatment with PARP inhibitors. Patients received rucaparib 600 mg twice daily for 12–14 days. Baseline bloods (EDTA and STRECK) and core biopsies (FFPE and RNAlater™) were collected at time of diagnostic biopsy or following trial entry. End of treatment bloods (STRECK) and biopsies (FFPE and RNAlater™) were taken at surgery or prior to neo-adjuvant chemotherapy within 24–48 h of the last rucaparib dose. The primary end point was Ki67 response from baseline to end of treatment defined as a ≥50% decrease. Secondary endpoints (pre-specified endpoints in the trial protocol) were association between baseline biomarkers of *BRCA1* methylation and a genomic predictor of HR deficiency (HRdetect) with Ki67 response to rucaparib, apoptosis induction following 12–14 days of rucaparib, the proportion of sporadic TNBC that fail to induce RAD51 foci on end of treatment biopsy (RAD51 score), safety and tolerability of rucaparib, association of biomarkers with RAD51 score, the proportion of patients with a change in circulating tumour DNA levels in response to rucaparib, association between change in circulating tumour DNA levels with biomarkers, and proportion of patients with *BRCA1* and *BRCA2* germline mutation related cancers with a Ki67 response to rucaparib and the proportion that have reduced RAD51 score and increased apoptosis induction. Pathological complete response was not assessed as an end point, as the two weeks window was considered to be too short for this end point. Where molecular analysis was not pre-specified in the trial protocol, this is highlighted as being ad hoc analsyis.

### Ki67 IHC and Scoring

Immunohistochemistry for Ki67 was performed and scored according to Leung et al^46^. Tissue sections were deparafinised and rehydrated prior to antigen retrieval using low pH (pH 6.0) Target Antigen Retrieval Solution (K8005, Dako UK Ltd). Tissue sections were stained for Ki67 using mouse monoclonal anti-Ki67 antibody (MIB-1 clone, Dako, M7240) diluted 1:50 in EnVision Antibody Diluent (K8006, Dako UK Ltd). Sections were washed using wash buffer (Dako, S3006) before colour development using REAL Kit (Dako, K5001). Tissues sections were counter stained with hematoxylin and coverslips mounted using DPX. The percentage of Ki67 positive cells was calculated from between 200-400 invasive tumour cells according to the method of Leung et al^46^. A Ki67 response was determined as a >50% fall in Ki67 at EOT compared to baseline.

### Cleaved PARP IHC and scoring

IHC for cleaved PARP (cPARP) was performed using a rabbit monoclonal antibody specific for PARP cleaved at Asp214. Tissue sections were deparafinised and rehydrated prior to antigen retrieval using High pH (pH9.0) Target Antigen Retrieval Solution (K8004, Dako UK Ltd), in PT-LINK (PT101, Dako UK Ltd). Tissue sections were stained for cPARP using rabbit monoclonal anti-cPARP antibody (Asp214, clone D64E10, Cell Signaling Technology, #5625) diluted 1:100 in EnVision Antibody Diluent (K8006, Dako UK Ltd). Sections were washed using wash buffer (Dako,S3006) before colour development using anti-rabbit link reagent EnVision FLEX LINKER (K8019, Dako UK Ltd). Tissues sections were counter stained with hematoxylin and coverslips mounted using DPX. The percentage of cPARP positive cells was calculated from a minimum of 500 invasive tumour cells. If fewer than 500 invasive tumour cells were present the sample was recorded as insufficient invasive tumour (IIT).

### Sample processing

All samples were processed by the central laboratory as part of the RIO trial. Fresh tumour samples were collected in RNAlater™ tubes, processed within 24 h and stored at -80^o^C until required for extraction. Baseline biopsies were sectioned using a cryostat. One section was cut for H&E and 16 sections were cut and stained with Nuclear Fast Red (NFR). A second H&E section was cut at the end of the series. H&E sections were reviewed and marked by a pathologist for macro-dissection. If the baseline biopsy did not have tumour the EOT biopsy (day 12–14) was used for sequencing. DNA was extracted using the Qiagen DNeasy Blood and Tissue kit according to the manufacturer’s instructions. DNA was eluted into 200 μl buffer ATE and stored at -20^o^C before quantification. DNA was quantified on the Bio-Rad QX-200 ddPCR system running Quantasoft v1.7, using the *RPPH1* (RNaseP, cat# 4403328 ThermoFisher) reference assay to calculate copies/well and multiplying by the *c* value (3.3 pg), an estimate of the mass of a single haploid human genome.

Blood collected in STRECK preservation tubes at baseline (day 1 prior to treatment) and end of treatment were processed within 24 h of sample collection. Plasma and buffy coat was separated by centrifugation 1600g for 20 min and stored individually at −80^o^C until DNA extraction. For plasma extraction, up to 4mls of archived plasma was extracted using the automated MagMax Cell-Free DNA Isolation Kit (Thermo Cat # A29319) and ThermoScientific KingFisher Flex Purification System. DNA was eluted into 100 μl buffer AVE and stored at −20^o^C. Buffy coat extraction was performed using the Qiagen DNeasy blood and tissue kit as per manufacturer’s instructions. DNA was eluted into 100 μl buffer AE and stored at −20 °C. Plasma DNA was quantified on the Bio-Rad QX-200 ddPCR system using the *RPPH1* reference assay to calculate copies/well and multiplying by the *c* value (3.3 pg), an estimate of the mass of a single haploid human genome.

### HRDetect Assay

Extracted DNA from fresh tissue with >20% tumour content and >200 ng quantifiable DNA, along with paired buffy coat germline DNA, were subject to whole-genome sequencing at the Sanger Institute, Cambridge, UK.

A 500-bp insert genomic libraries were constructed according to Illumina library protocols and 150 bp paired-end sequencing performed on an Illumina HiSeq X Ten using HCS (v3.5.0) for HiSeq X systems, to an average sequence depth of 38.5× for both tumour and normal. The resulting reads were aligned to the reference human genome (GRCh37) using Burrows-Wheeler Aligner (BWA) (0.7.16a (r1181)). Mutation calling was performed as described previously^12^. CaVEMan (Cancer Variants Through Expectation Maximization: http://cancerit.github.io/CaVEMan/) was used for calling somatic substitutions. Indels in the tumour and normal genomes were called using a modified Pindel version 2.0. (http://cancerit.github.io/cgpPindel/). Structural variants were discovered using a bespoke algorithm, BRASS (BReakpoint AnalySiS) (https://github.com/cancerit/BRASS). All annotation was to Ensembl build 75. Allele-specific copy number analysis of tumours was performed using ASCAT (v2.1.1) applied to next-generation whole-genome sequencing data as described previously^11,12^. Copy number values and estimates of aberrant tumour cell provided by ASCAT were input into the CaVEMan substitution algorithm. In addition, ASCAT segmentation profiles were used to establish the presence of copy number changes and loss of heterozygosity across the *BRCA1*, *BRCA2* and *PALB*2 genes.

The predominant mutational signatures present in breast cancer have been identified in a large WGS study involving 560 breast cancers. These comprise 12 substitution signatures and 6 structural rearrangement signatures. The contributions of these consensus mutational signatures were estimated in the 27 RIO trial WGS samples as described previously^12,47^. In addition, the contribution of small insertions and deletion at regions of micro-homology or repeats and HRD LOH index were estimated^7^.

Mutational signature contributions for substitution signatures 3 and 8, rearrangement signatures 3 and 5, deletions at microhomology and HRD LOH index were calculated for each sample as input in to the weighted model, HRDetect. The HRDetect algorithm was run as described previously, using the previously described weights^11^.

### Targeted tissue sequencing

Paired tissue and buffy coat DNA were sent to the Centre of Molecular Pathology at The Royal Marsden Hospital for sequencing using a targeted capture-based approach designed to detect mutations and amplifications frequently seen in breast cancer. The targeted panel, “ABC-Bio” panel, has been validated in the ABC-Bio (molecular screening for patients with advanced breast cancer) trial and comprises of 41 genes commonly mutated in breast cancer^48^. Libraries were run on a MiSeq (Illumina) using MiSeq Reporter (MSR v2.5.1). All internally developed code is accessible on request.

### AVENIO sequencing

ctDNA samples from six patients from whom adequate tumour samples were not obtained were sent to Roche for sequencing using the AVENIO ctDNA targeted tumour profiling kit (Roche Sequencing). A total of 4 samples were sequenced on the AVENIO ctDNA targeted kit (17 genes) and 2 samples were run on the AVENIO ctDNA expanded kit (77 genes), using HighOutput 300 cyc kit on a NextSeq 500 (Illumina) using NextSeq system suite (v2.2.0). Data was analysed using the AVENIO Oncology analysis software (v1.0.0 and v1.1) available from Roche.

### BRCA1 and Rad51C methylation, bisulfite sequencing

The promoter region of *BRCA1* and *RAD51C* was identified using the Eukaryotic Promoter Database (http://epd.vital-it.ch/index.php). *BRCA1* promoter was amplified with forward-TATTTTGAGAGGTTGCTGTTTAG and reverse-CTAAAAAACCCCACAACCTATCCC primers. Analysis of BRCA1 methyaltion was pre-planned, and *RAD51C* methylation was added ad hoc to *BRCA1* methylation prior to association with activity surrogates, as the potential importance of *RAD51C* methylation in TNBC was only recognised after the start of the trial^2^. The *RAD51C* promoter was amplified with forward-TGGTAATTGGTTAGTGTGTGT and reverse-TCCTCATCAAATATACACCCTAACT primers. *BRCA1* and *RAD51C* PCR conditions were optimised for multiplex assay using ThermoFisher Scientific AccuPrime Hi-fidelity Taq. Human methylated and unmethylated DNA (Zymo Research, Human HCT116DKO non-methylated DNA and HCT116DKO methylated DNA) primers were used as a control.

Extracted DNA from RIO RNA Later samples were subjected to bisulfite sequencing (Zymo Research Methylation Gold spin column kit D5005). Total DNA input ranged from 10 to 500 ng. Samples were quantified post bisulfite sequencing using Qubit 3.0 fluorimeter and subsequently subjected to PCR using ThermoFisher Scientific AccuPrime Hi-fidelity Taq at 60 °C for 34 cycles. Samples were cleaned using Qiagen QIAquick PCR purification kit (ID:28104) and quantified using Qubit 3.0 fluorimeter. Samples were subjected to Illumina NebNext Ultra II library preparation. Total library input ranged from 10-116 ng and PCR cycles were adjusted accordingly, and sequenced on the Illumina MiniSeq platform with Miniseq system suite v1.1. Mean number of reads for BRCA1 amplicon was 36909 (range 9654-60084) and RAD51C amplicon 48879 (range 28404- 71129) with a mean 47% of reads on target for the methylation sequencing run (range 37-50%).

Bioinformatics analysis of methylation followed a similar workflow to previous studies^32^. Paired overlapping reads were merged into a single sequence using flash^49^ after adaptor trimming using trim-galore (https://www.bioinformatics.babraham.ac.uk/projects/trim_galore/). Each read was aligned using pairwise alignment to the BRCA1 or RAD51C amplicons using Biostrings R^50^ package with 90% identity. Reads with more than 1 mismatch in alignment were additionally removed. Reads with incomplete bisulphite conversion were removed by calculating the unconverted cytosine count at non CG sites as well as reads where all CG sites in the read were not C or T. Reads were assessed as being methylated when >90% of CpG sites in the amplicon were methylated. For RAD51C methylation, 2 sites in the RAD51C amplicon were removed as these were found to be consistently methylated in all samples.

### Immunohistochemistry for RAD51/Geminin (GMNN) double staining

FFPE samples taken 24–48 h after the last dose of rucaparib were cut, deparafinised, rehydrated and stained with hematoxylin and eosin (H&E) and double stained with GMNN and RAD51. Pre- and post-radiotherapy-induced squamous cell carcinoma were used as negative and positive controls.

Antigen retrieval was performed and RAD51 primary antibody (mouse monoclonal, Genetex, GTX70230) was diluted 1/200 in Dako antibody diluent (K8006) and applied. Slides were incubated with Dako Envision Flex HRP (K8002). Geminin (GMNN) antibody (rabbit polyclonal, Proteintech 10802-1-AP) diluted 1/1500 in Dako antibody diluent was applied and incubated with Dako Envision Flex HRP (K8002). Sections incubated with Vector TMB blue (SK4400) and counterstained with Gills 1 hematoxylin, air dried and dehydrated in xylene before mounted and cover slipped with Vectamount.

Five random fields at 40× magnification were identified and marked in PathXL. GMNN staining was identified with blue/green staining and RAD51 was identified by the presence of brown nuclear foci. Scoring was done by 2 scorers blinded to each other, time point and clinical details.

The number of tumour cells, GMNN-positive cells and RAD51-positive cells were counted. Cells with 5 or more RAD51 foci were classified as RAD51 positive. A minimum of 300 tumour cells and 30 GMNN-positive cells were a minimum requirement for inclusion. Raw data were collected and the proliferation fraction ((no. of GMNN-positive cells / total number of tumour cells) × 100) and RAD51 score ((no. of RAD51-positive cells / no. of GMNN-positive cells) × 100) was calculated.

### Circulating tumor DNA analysis

For each trial subject, dPCR assays were designed for the mutations identified by tissue sequencing according to the method of Garcia-Murillas et al^22^. Assays were optimised with temperature gradients and patients with more than one mutation had multiplex assays optimised. If optimisation was not achieved with multiplexes, a singleplex assay was used.

Mutation analysis was done using ddPCR assays specific for each patient’s mutation(s) on a QX200 system (Bio-Rad) running Quantasoft v1.7. 50 μl DNA (2 ml plasma equivalent) was used divided equally into 2 wells from each time point. DNA was dried at 60 degrees for 100 min before preparing the PCR reactions to a volume of 20 μl. Three NTCs and a negative control of the patient’s buffy coat DNA were included for each dPCR assay. Only paired samples with at least 4 positive droplets in baseline samples were analysed for change in ctDNA levels. The circulating DNA ratio at day 15 (CDR15) was assessed as a ratio of the ctDNA copies/ml at EOT copies/ml compared to ctDNA copies/ml at baseline. Where more than one mutation was tracked a weighted mean of ctDNA change was calculated.

The CDR15 cutoff <0.25 for ctDNA suppression, was pre-specified determined in a separate study in metastatic breast cancer, that validated this cutoff to predict progression free survival on cytotoxic paclitaxel therapy^25^.

### RNA sequencing

Paired RNAlater™ samples were identified and sectioned using a cryostat. One section was cut for H&E and 10 sections were cut and stained with nuclease-free Nuclear Fast Red. A second H&E section was cut at the end of the series. H&E sections were reviewed and marked by a pathologist for tumour and assessed for tumour content. NFR stained sections were micro-dissected and RNA was extracted using the Qiagen RNeasy Mini kit according to the manufacturer’s instructions. RNA was eluted twice into separate 50 μl RNA free water and stored at -80^o^C before quantification. RNA was quantified using Qubit 3.0 fluorimeter using the Qubit™ RNA HS Assay Kit (Q32852, ThermoFisher Scientific).

Extracted RNA (~1 μg) was sent to Eurofins Genetic Services Limited for RNA Exome sequencing. Total RNA was subjected to RiboZero depletion and Illumina TruSeq RNA Exome library preparation. Libraries were pooled and sequenced on an Illumina HiSeq 2500 (v4 chemistry) running HiSeq Control Software (HCS) v2.2.68. Samples were aligned to the GChr37 genome using STAR aligner (https://github.com/alexdobin/STAR)^51^ with a mean of 44,117,169 reads per sample (range 14010752-91,470,225). Gene counts were established using htseq (https://github.com/simon-anders/htseq)^52^. DeSeq2 (10.18129/B9.bioc.DESeq2)^53^ was used to establish gene-wise normalisation and to look for differential expression between different sample groups. Gene set enrichment analysis was carried out using the R package fgsea (10.18129/B9.bioc.fgsea)^54^. PAM50 and TNBC subtypes were established using AIMS (10.18129/B9.bioc.AIMS)^55^ and TNBCtype (http://cbc.mc.vanderbilt.edu/tnbc/)^56^. Cibersort (https://cibersort.stanford.edu/index.php)^29^ was run in absolute mode using normalized gene counts.

### Statistics

The study size was determined using a Simon two stage Minimax design on Ki67 response in patients with sporadic TNBC, with p0 = 10% and p1 = 25% Ki67 response rate. With a two-sided alpha 1.6% and 90% power, four Ki67 responders were required in the first 41 assessable patients to proceed to a full 73 patients. The study would declare inefficacy if <4/41 or <14/73 responses were observed. An initial futility assessment was also planned after 20 evaluable patients had completed rucaparib treatment and consideration would be given to stopping the trial if 0 responses were observed. Additionally, 5, 1.6 and 1.6% two-sided alphas were allocated to assess rucaparib activity within BRCA1 methylated tumours, RAD51 foci formation tumours, and genomic classifier HRDetect tumours respectively, for a total study two-sided alpha of 10%. Up to 20 patients with known *BRCA1* or *BRCA2* pathogenic germline mutations at the time of trial entry were recruited as controls for exploratory determination of biomarker endpoints. The study closed to new patients after 43 patients had been recruited on advice of the IDMC (Fig. 1) due to low recruitment.

Analysis of response data was performed on patients who had taken rucaparib for 7 days or more. Safety and tolerability are assessed in all patients who received at least one dose of rucaparib. Response rates and proportions are reported with 95% confidence intervals. Associations between biomarkers are analysed using Fisher’s exact test or Mann–Whitney as appropriate. Change in biomarkers between baseline and day12–14 samples are analysed using the Wilcoxon signed rank test. Analyses were conducted in Stata v13 and GraphPad Prism, with all analyses reported two-sided.

### Reporting summary

Further information on research design is available in the Nature Research Reporting Summary linked to this article.

## Supplementary information

Supplementary Information

Reporting Summary

## Data Availability

Sequencing data from whole-genome sequencing, exome RNA seq, and targeted sequencing from tumour samples that support the findings of this study (Figs. 1–5 and Supplementary Fig. 1, 3 and 4) is deposited in the European Genome-phenome Archive (EGA), reference EGAS00001004405 https://ega-archive.org.

## Code availability

The code used for the analysis of sequencing data is detailed here with links and references where appropriate.

Whole-genome sequencing: CaVEMan (Cancer Variants Through Expectation Maximization: http://cancerit.github.io/CaVEMan/), Pindel version 2.0. (http://cancerit.github.io/cgpPindel/), BRASS (BReakpoint AnalySiS) (https://github.com/cancerit/BRASS), and ASCAT (v2.1.1). Annotation was to Ensembl build 75. Code for targetted tumour sequencing is available upon request. AVENIO ctDNA sequencing: AVENIO Oncology analysis software (v1.0.0 and v1.1) available from Roche. Bisulfite sequencing: Eukaryotic Promoter Database (http://epd.vital-it.ch/index.php), trim-galore (https://www.bioinformatics.babraham.ac.uk/projects/trim_galore/), Biostrings R package. RNA sequencing: STAR aligner (https://github.com/alexdobin/STAR), htseq (https://github.com/simon-anders/htseq), DeSeq2 (10.18129/B9.bioc.DESeq2), fgsea (10.18129/B9.bioc.fgsea), AIMS (10.18129/B9.bioc.AIMS), TNBCtype (http://cbc.mc.vanderbilt.edu/tnbc/) and Cibersort (https://cibersort.stanford.edu/index.php).
