# Supplementary material for: Homologous recombination DNA repair deficiency and PARP inhibition activity in primary triple negative breast cancer
Source: Nat Commun. 2020 May 29;11:2662. doi: 10.1038/s41467-020-16142-7 (PMC7260192; doi:10.1038/s41467-020-16142-7)
Supplement: Supplementary file 1 — Supplementary Information [file 41467_2020_16142_MOESM1_ESM.pdf]

SUPPLEMENTARY FIGURES

Supplementary Figure 1. HRDetect analysis

A

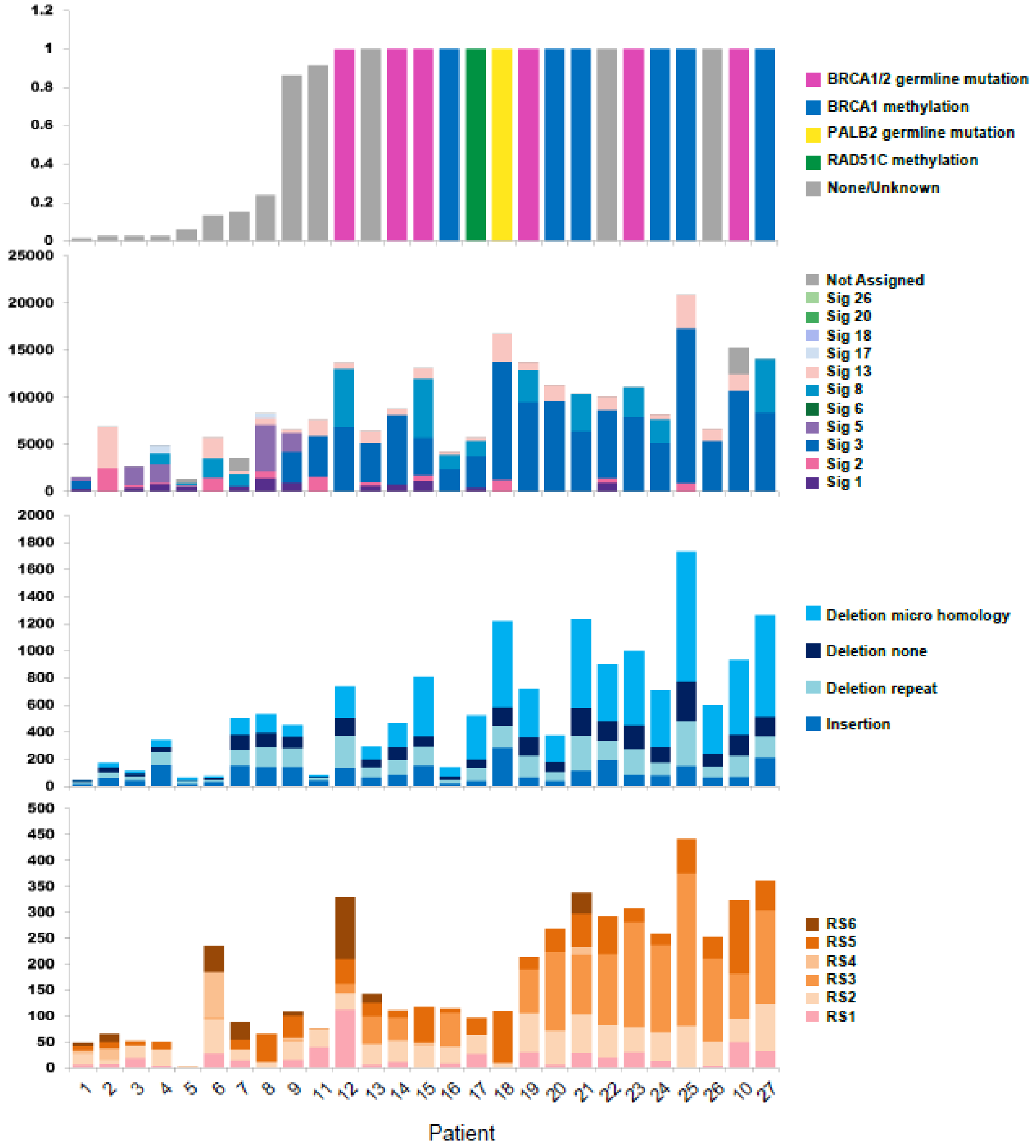

B

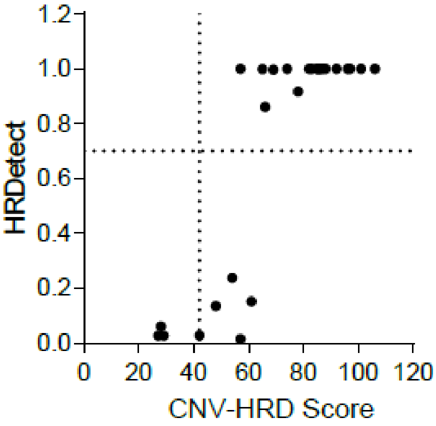

A. Summary of the genomic characteristics of WGS samples. Histograms show from top to bottom; HRDetect score, samples are ordered by ascending HRDetect score across the x axis from left to right. Coloured bars indicate inactivating mutations and promoter methylation of HR genes. Next, contribution of substitution signature; indel types; contribution of rearrangement signatures.

B. Association of copy number based HRD index and HRDetect score. Thresholds for both assays are indicated by dotted lines.

Supplementary Figure 2. RAD51 focus IHC assay validation in the chemoNEAR study

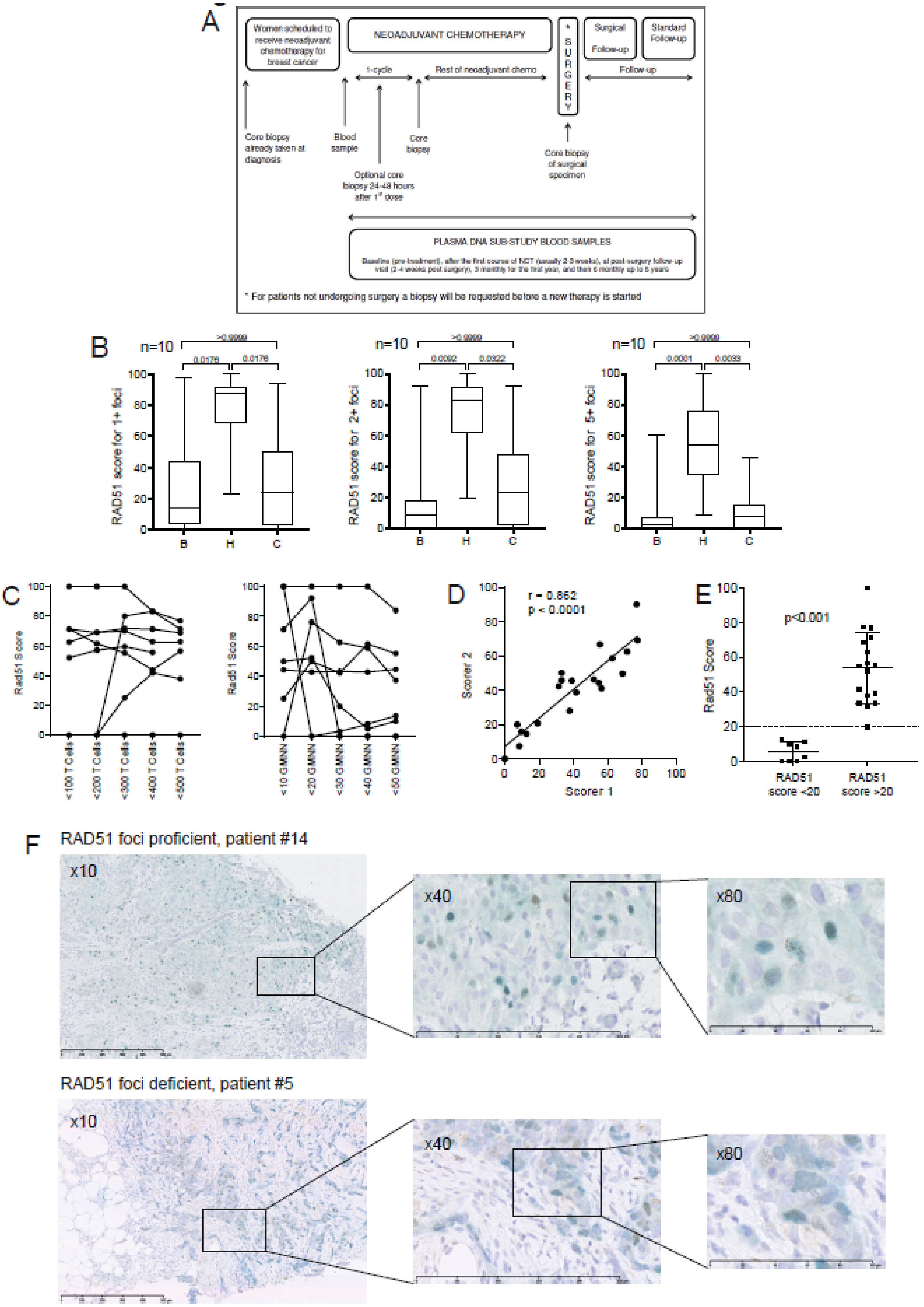

A. Trial Schema of ChemoNEAR Study (CCR3449, REC ID: 11/EE/0063),a multicentre biological research study involving all patients irrespective of hormone receptor/ HER2 status undergoing neoadjuvant chemotherapy for primary breast cancer. Research biopsies were collected at baseline "B", 24-48 hours post 1st cycle of epirubicin and cyclophosphamide chemotherapy "H" (optional) and prior to the 2nd cycle "C". Samples were processed at local centres by formalin fixation and paraffin embedding (FFPE) before being sent to and stored at The Royal Marsden Hospital. Patients who had the optional 24-48 hour biopsy were identified and their corresponding baseline and 2nd cycle core biopsies were retrieved for RAD51 analysis. All patients analysed had the optional biopsy taken at 24 hours.

B. RAD51 scoring in 10 sets of paired biopsies assessed using either 1+ foci (Left), 2+foci (Centre) or 5+ foci (Right) per geminin (GMNN) positive tumour cell, at baseline (B), 24-48 hours post 1st cycle of chemotherapy (H) and prior to the 2nd cycle of chemotherapy (C), demonstrating 5+ RAD51 foci/cell to be the most robust assessment of RAD51 scoring. RAD51 foci were only assessed in tumor cells that expressed geminin (GMNN). Centre line, median; box, interquartile range and bars, 95%CI. Statistical analysis with non-parametric Friedman test and Dunn's multiple comparisons test, p values as indicated.

C. Eight samples were used to determine the minimum requirements for RAD51 scoring. RAD51 score based on the number of tumour cells (Left) and GMNN +ve cells (Right) counted in the sample. RAD51 scores were found to be robust and consistent with minimum requirements for an acceptable RAD51 score set at >300 tumor cells and >30 GMNN +ve cells.

D. Correlation between scorers of RAD51 IHC using 5+foci for independent analysis by two different readers (scorers). Spearman correlation; n=21, r=0.862, p<0.0001.

E. RAD51 IHC score in 19 baseline cancer biopsies and 25 cancer biopsies taken 24 hours after chemotherapy. RAD51 IHC cut-off score of 20 (line) robustly identified DNA damage induced RAD51 foci induction, with 5% (1/19) of baseline samples >20% and 84% (12/24) of 24 hour biopsies >20% (p<0.0001).

F. Examples of RAD51/geminin dual Immunohistochemistry showing tumours deficient in RAD51 foci (top panel) and tumours proficient in RAD51 foci (bottom panel) at low and high power.

Supplementary Figure 3. CIBERSORT analysis of tumour biopsies on RIO study.

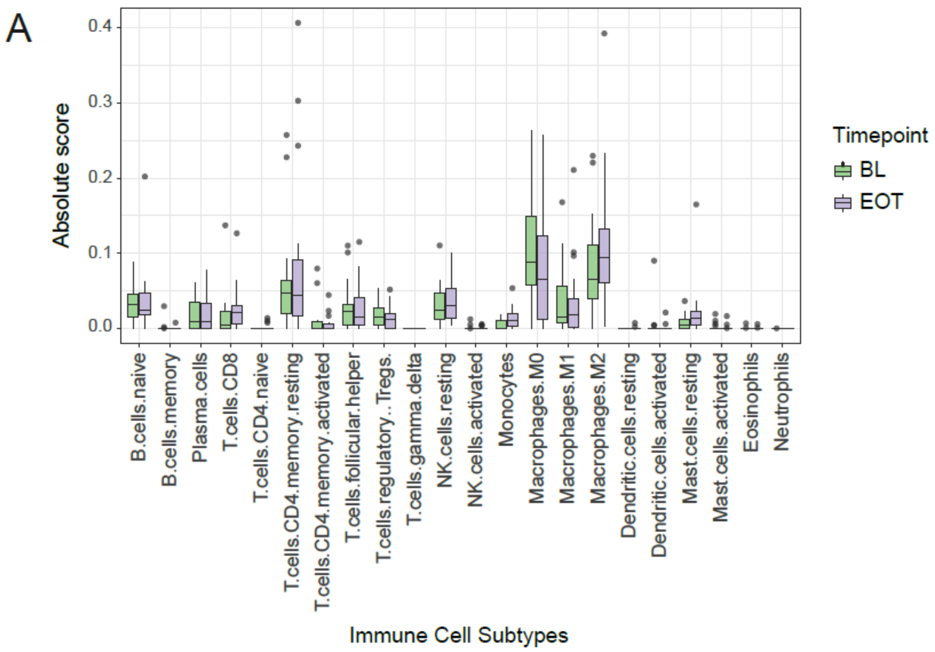

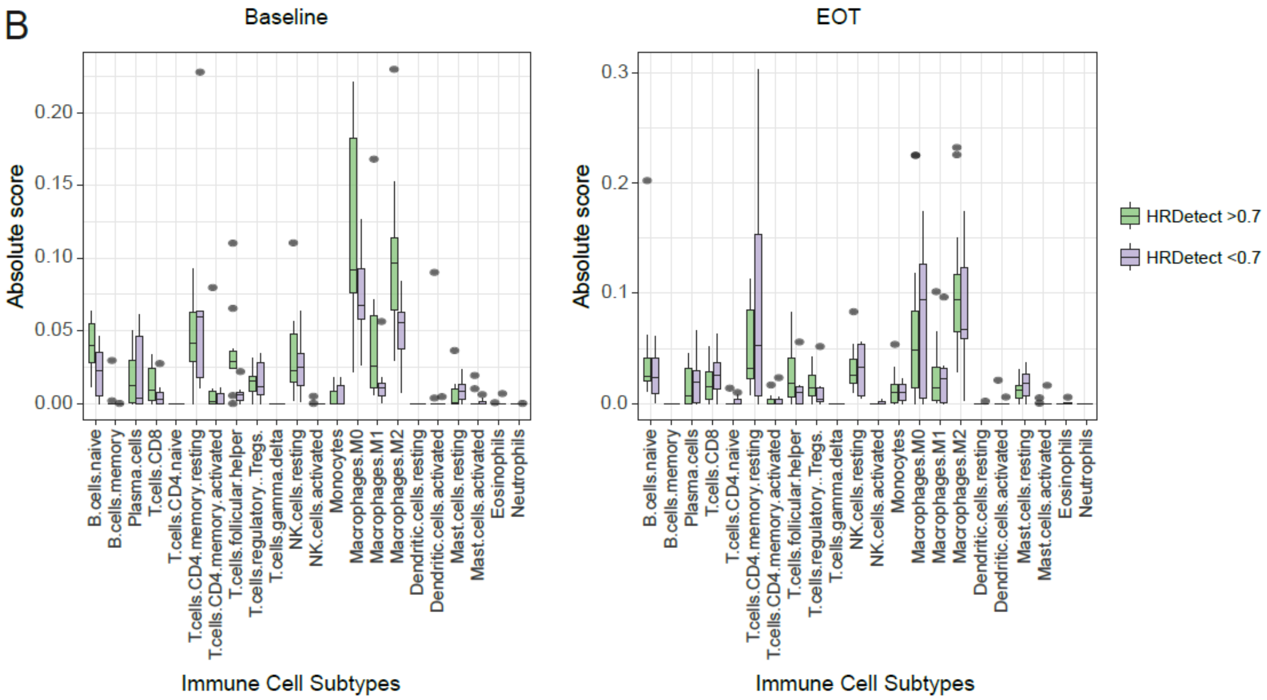

A. CIBERSORT analysis of immune cell subtypes in all samples presented by time point.

B. CIBERSORT analysis of immune cell subtypes in HRDetect score positive (>0.7) and negative (<0.7) samples presented by baseline (Left) and EOT (Right).

Centre line, median; box, interquartile range; and bars, highest /lowest point within 1.5 interquartile range.

Supplementary Figure 4. Gene expression pathway in cancers HR Detect positive and negative

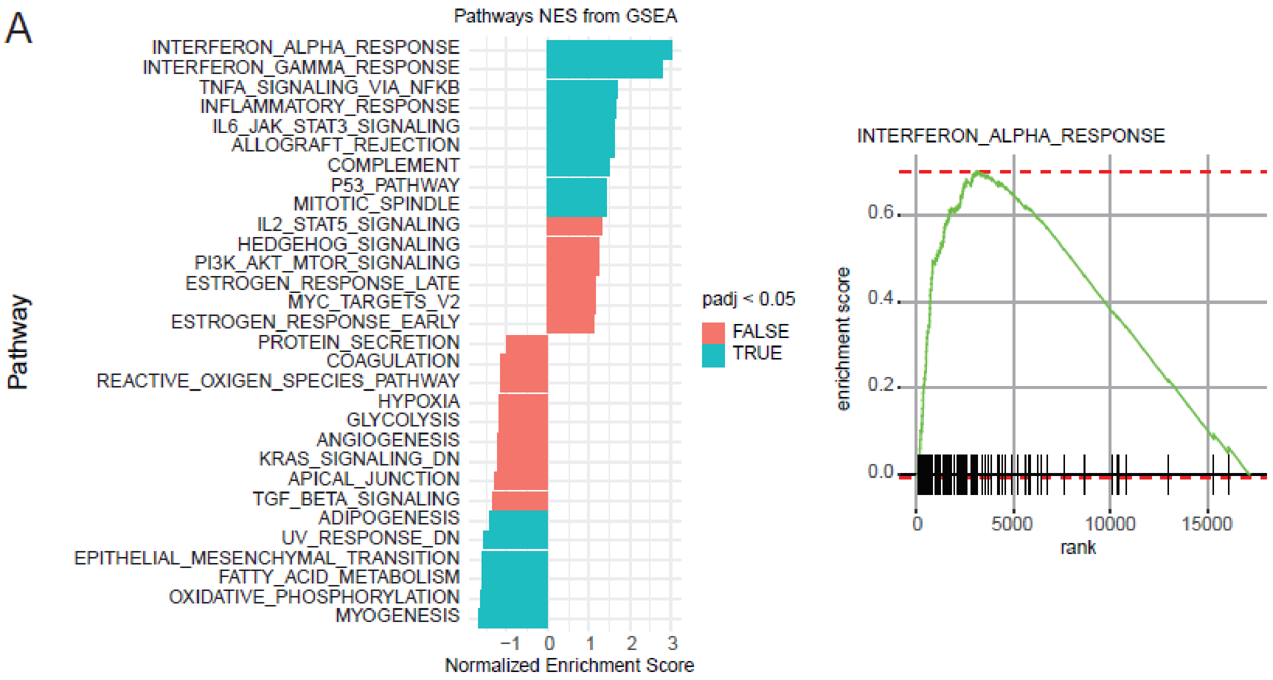

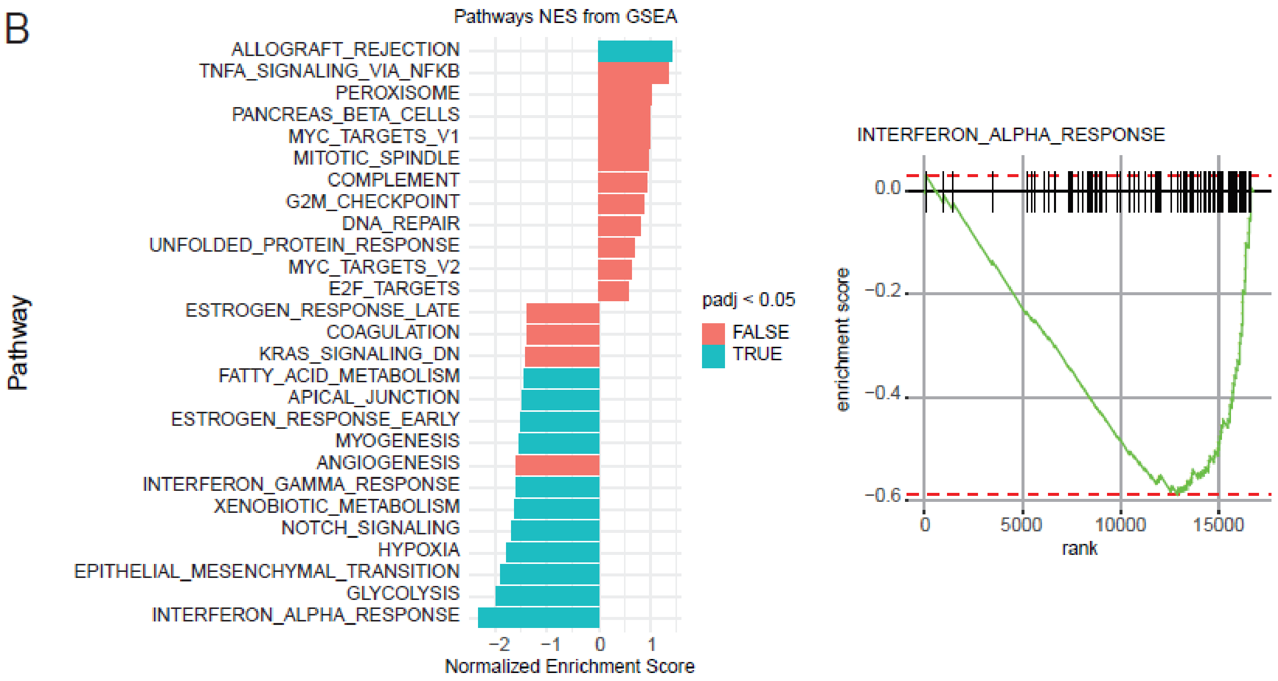

A. *Left,* Gene set enrichment pathway analysis (GSEA) for gene expression changes through treatment in HRDetect positive cancers (n=11 paired tumor samples). *Right*, increased expression of interferon-α pathway genes on rucaparib, q=0.003. False discovery rate corrected q value for change.

B. *Left,* Gene set enrichment pathway analysis (GSEA) for gene expression changes through treatment in HRDetect negative cancers (n=6 paired tumor samples). *Right*, decreased

expression of interferon-α pathway genes on rucaparib, q=0.003. False discovery rate corrected q value for change.

SUPPLEMENTARYTABLES

Supplementary Table 1. Adverse events reported in RIO study in at least 10% patients.

Preferred Term	Any Grade		Grade3		Grade4	
	n	%	n	%	n	%
Fatigue	30	71.4	1	2.4	0	0.0
Liver function test increased	29	69.0	5	11.9	0	0.0
Nausea	28	66.7	0	0.0	0	0.0
Hypertension	21	50.0	2	4.8	0	0.0
Headache	19	45.2	1	2.4	0	0.0
Dyspepsia	13	31.0	0	0.0	0	0.0
Constipation	11	26.2	0	0.0	0	0.0
Diarrhoea	11	26.2	3	7.1	0	0.0
Lymphopenia	10	23.8	0	0.0	0	0.0
Dizziness	10	23.8	0	0.0	0	0.0
Dysgeusia	10	23.8	0	0.0	0	0.0
Vomiting	9	21.4	0	0.0	0	0.0
Hypercholesterolaemia	9	21.4	0	0.0	0	0.0
Decreased appetite	8	19.0	0	0.0	0	0.0
Anaemia	7	16.7	0	0.0	0	0.0
Leukopenia	6	14.3	0	0.0	0	0.0
Neutropenia	6	14.3	1	2.4	2	4.8
Blood bilirubin increased	6	14.3	0	0.0	0	0.0
Gamma-glutamyltransferase increased	6	14.3	0	0.0	0	0.0
Rash	6	14.3	0	0.0	0	0.0
Tachycardia	5	11.9	0	0.0	0	0.0
Asthenia	5	11.9	0	0.0	0	0.0
Muscular weakness	5	11.9	1	2.4	0	0.0

***All grade 3/4 cases of neutropenia occurred in patients receiving neoadjuvant chemotherapy during the 30 day post-treatment reporting window*

31 patients (72%) completed treatment per protocol with no interruptions

Supplementary Table 2. Individual patient data for analysis of HR deficiency with individual gene analysis, HR detect, HRD index, and RAD51 foci functional assessment.

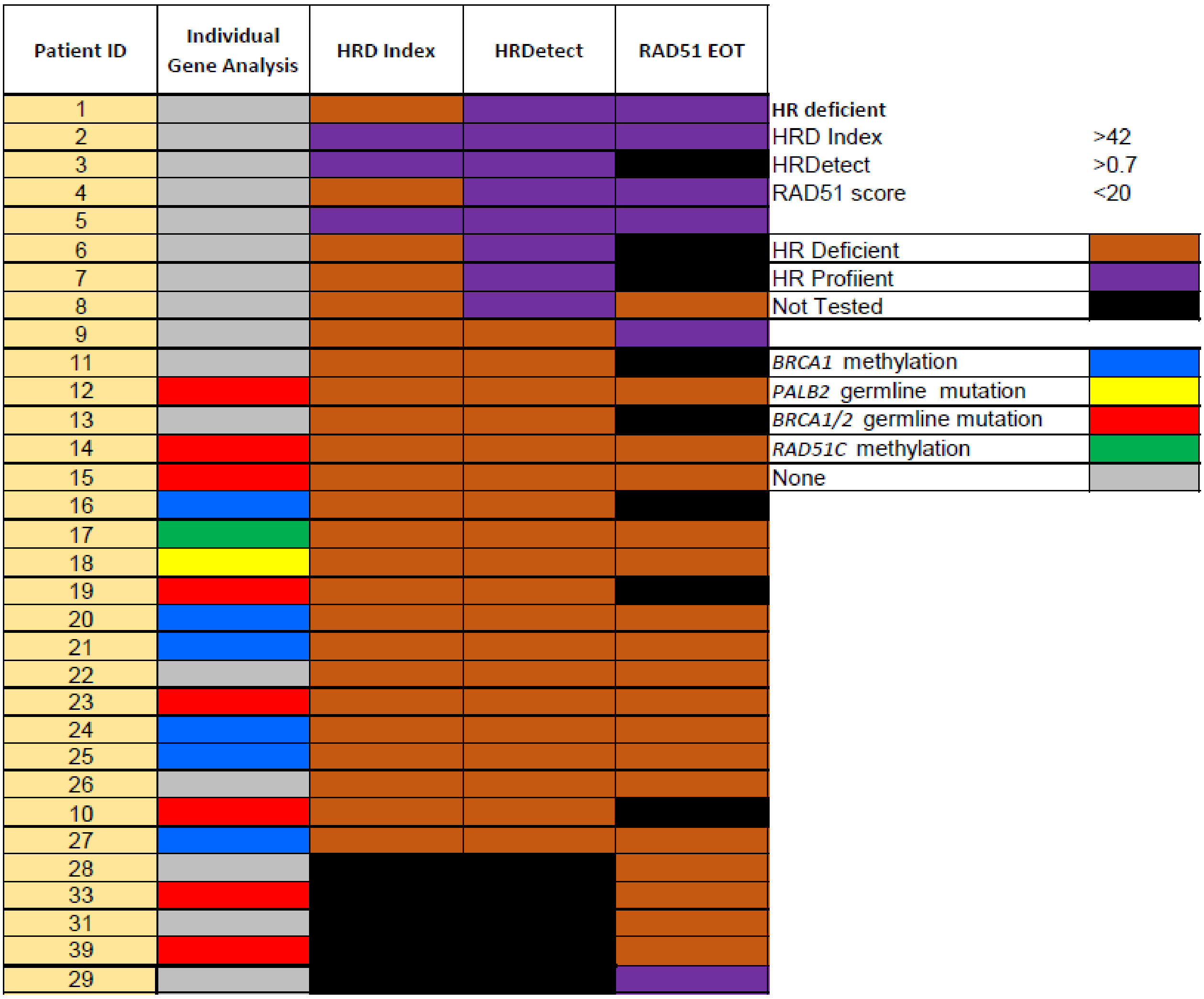

Supplementary Table 3. Mutations identified in tumor samples.

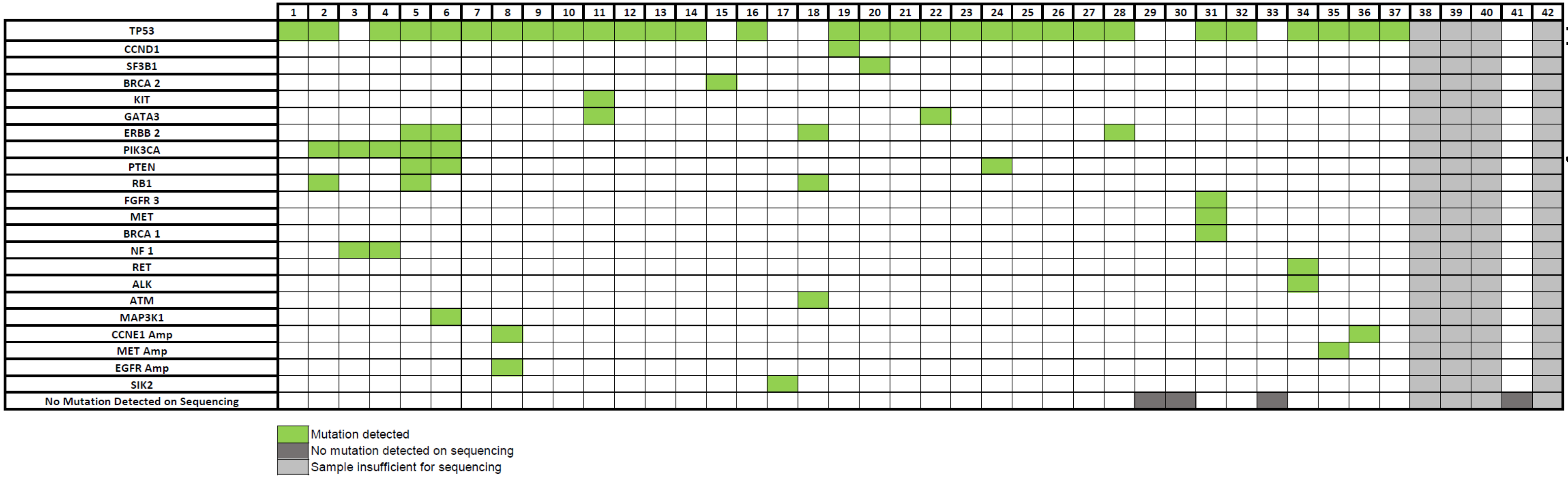

Mutation for DDPCR	No. detected	% frequency
TP53	30	45
PIK3CA	5	8
PTEN	4	6
ERBB 2	4	6
RB1	3	5
NF 1	2	3
GATA3	2	3
CCNE1 Amp	2	3
CCND1	1	2
SF3B1	1	2
BRCA 2	1	2
KIT	1	2
FGFR 3	1	2
MET	1	2
BRCA 1	1	2
RET	1	2
ALK	1	2
ATM	1	2
MAP3K1	1	2
MET Amp	1	2
EGFR Amp	1	2
SIK2	1	2
No Mutation Detected	4	6
Not sequenced	5	NA
Total (mutations only)	66	

Supplementary Table 4. Personalised ddPCR assays for mutation tracking in ctDNA.

Patient	Gene	Nucleotide Change	Seq Primer F	Seq Primer R	WT probe Sequence	5' modification	3' modification	Mutant Probe Sequence	5' modification	3' modification	ddPCR ann/ ext Temp (C)	Amplicon Length (bp)
1	TP53	c.560-1G>A	CCAGGCCTCTGATTCCTCACT	CCTTCCACTCGGATAAGATGCT	ATTGCTCTTAGGTCTG	HEX	Iowa Black FQ	ATTGCTCTTAAGTCTGG	6-FAM	Iowa Black FQ	53	69
2	TP53	c.659A>G	TTGGATGACAGAAACACTTTTCGAC	AGACCCCAGTTGCAAACCA	TGGTGCCCTATGAGCC	HEX	Iowa Black FQ	TGGTGCCCTGTGAGCC	6-FAM	Iowa Black FQ	52	79
2	PIK3CA	c.3140A>G	TGAGCAAGAGGCTTTGGAGT	TCAGTTCAATGCATGCTGTTT	AATGATGCACATCATGGTGGCT	VIC	NFQMGB	AATGATGCACGTCATGGTGGCT	6-FAM	NFQMGB	52	115
3	NF1	c.2409+1G>C	AACTATCCAAAAGCCAAAATGGAA	GCAGATCAGTTAACAGACAAAAGTCAA	TGGCCAGGTAAGTCTGTAA	HEX	Iowa Black FQ	TGGCCACGTAAGTCTGTAA	6-FAM	Iowa Black FQ	52	73
4	TP53	c.814G>T	GCTTCTCTTTTCCTATCCTGAGTAGTG	CCCAGGACAGGCACAAACA	AACAGCTTTGAGGTGC	HEX	Iowa Black FQ	AACAGCTTTGAGTTGC	6-FAM	Iowa Black FQ	52	77
5	TP53	c.972_975delTGGA	CTCTCCCCAGCCAAAGAAGA	AACGGCATTTTGAGTGTTAGAC	ACTGGATGGAGAATAT	HEX	Iowa Black FQ	ACTGGAGAATATTTC	6-FAM	Iowa Black FQ	52	115
5	ERBB2	c.2264T>C	AGGGCATCTGGATCCCTGAT	CGTCTAAGATTTCTTTGTTGGCTTTGG	ATCAAAGTGTTGAGGGA	HEX	Iowa Black FQ	TGGCCATCAAAGTGTCGA	6-FAM	Iowa Black FQ	52	102
5	PIK3CA	c.3140A>G	TGAGCAAGAGGCTTTGGAGT	TCAGTTCAATGCATGCTGTTT	AATGATGCACATCATGGTGGCT	VIC	NFQMGB	AATGATGCACGTCATGGTGGCT	6-FAM	NFQMGB	52	115
6	TP53	c.713G>T	TTGGCTCTGACTGTACCACCAT	CCGCCCATGCAGGAACT	CACTACAACTACATGTGTAA	HEX	Iowa Black FQ	CACTACAACTACATGTTTA	6-FAM	Iowa Black FQ	52	61
7	TP53	c.421T>C	CTCAACAAGATGTTTTGCCAACTG	GGGTGTGGAATCAACCCACA	CAAGACCTGCCCTGTG	HEX	Iowa Black FQ	CAAGACCCGCCCTGT	6-FAM	Iowa Black FQ	52	66
9	TP53	c.715A>G	TCTGACTGTACCACCATCCACTACA	CATGCCGCCCATGCA	CTACATGTGTAACAGTTC	HEX	Iowa Black FQ	CATGTGTGACAGTTC	6-FAM	Iowa Black FQ	52	60
10	TP53	c.659A>G	TTGGATGACAGAAACACTTTTCGAC	AGACCCCAGTTGCAAACCA	AGACCCCAGTTGCAAACCA	HEX	Iowa Black FQ	TGGTGCCCTGTGAGCC	6-FAM	Iowa Black FQ	52	79
11	TP53	c.993+1T>G	GAAGAAACCACTGGATGGAGAATATT	CCACTTGATAAGAGGTCCCAAGA	CACCCTTCAGGTAC	HEX	Iowa Black FQ	CACCCTTCAGGGACT	6-FAM	Iowa Black FQ	52	67
12	TP53	c.715A>G	TCTGACTGTACCACCATCCACTACA	CATGCCGCCCATGCA	CTACATGTGTAACAGTTC	HEX	Iowa Black FQ	CATGTGTGACAGTTC	6-FAM	Iowa Black FQ	52	59
13	TP53	c.637C>T	TTTGCGTGTGGAGTATTTGGAT	CTCATAGGGCACCACCACACT	ACAGAAACACTTTTCGAC	HEX	Iowa Black FQ	ACAGAAACACTTTTTGAC	6-FAM	Iowa Black FQ	53	63
14	TP53	c.323_331delGTTTCCGTCinsTG	TGTCCCTTCCCAGAAAACCT	CAGGCATTGAAGTCTCATGGA	TTTCCGTCTGGGCTTCTTG	HEX	Iowa Black FQ	ACGTGTGGGCTTCTTGCAT	6-FAM	Iowa Black FQ	52	133
15	BRCA2	BRCA2 (Sub + Del)	GCATTGGAGGAATATCGTAGGT	GTCAATACCTGCTTTGTTGCAG	CATTTTTTGAAATTTTTAAGACAC	HEX	Iowa Black FQ	CATTTTTAGAAATTTTAAGACAC	6-FAM	Iowa Black FQ	52	70
16	TP53	c.665delC	TGGATGACAGAAACACTTTTCGA	GCCACTGACAACCACCCTTAAC	CCCTATGAGCGCCT	HEX	Iowa Black FQ	CCCTATGAGC~~C~~GCC	6-FAM	Iowa Black FQ	52	112
17	SIK	c.1189G>C	TGAGGCTGCTGCGATCTG	GCAGCTTCCGCCTGACA	TCCAACGTGGAGGC	HEX	Iowa Black FQ	CCAACGTGCAGGC	6-FAM	Iowa Black FQ	52	87
18	ATM	c.8293G>A	GCTGAATGATCATCAAATGCTCTTT	TCACCAATGGGGACAGTTCC	CAGCGAAGTGGTGTTCTTGA	HEX	Iowa Black FQ	CAGCGAAGTAGTGTTCTTGA	6-FAM	Iowa Black FQ	57.5	133
19	CCND1	c.433G>C	CCCTGTGTTCGCAGCAAAT	CGGCCAGGTTCCACTTGA	AGCTGCTCCTGGTG	HEX	Iowa Black FQ	TGCTCCTGCTGAACA	6-FAM	Iowa Black FQ	52	60
19	TP53	c.773A>G	AACCGGAGGCCCATCCT	TGTGCAGGGTGGCAAGTG	CATCACACTGGAAGAC	HEX	Iowa Black FQ	CATCACACTGGGAGAC	6-FAM	Iowa Black FQ	52	70
20	SF3B1	c.3028A>G	GTGAACAAAAGTTGCAATTCAAATG	GGTGAGTCTAGGCAGCAGATCTTTA	ATGCATAAGATGACTCC	HEX	Iowa Black FQ	ATGCATAAGATGGCTC	6-FAM	Iowa Black FQ	52	103
20	TP53	c.713G>A	TTGGCTCTGACTGTACCACCAT	CCGCCCATGCAGGAACT	CACTACAACTACATGTGTAA	HEX	Iowa Black FQ	CACTACAACTACATGTATAA	6-FAM	Iowa Black FQ	52	60
21	TP53	c.596G>T	GGCCCCTCCTCAGCATCT	ATCCAAATACTCCACACGCAAA	TCCGAGTGGAAGGAAATTT	HEX	Iowa Black FQ	TCCGAGTGGAAGTAA	6-FAM	Iowa Black FQ	52	57
22	GATA3	c.829C>T	AGAAGGCAGGGAGTGTGTGAA	CAGGCGTTGCACAGGTAGTGT	ACTGTGGCGGCGAG	HEX	Iowa Black FQ	ACTGTGGCGGTGAGA	6-FAM	Iowa Black FQ	53	64
22	TP53	c.1024delC	GGCGTGAGCGCTTCGA	CAGCCTGGGCATCCTTGA	ATGTTCCGAGAGCTGAAT	HEX	Iowa Black FQ	ATGTTCGAGAGCTGAATGA	6-FAM	Iowa Black FQ	53	66
23	TP53	c.356_360delCCAAG	GTCTGGGCTTCTTGCATTCT	GCCAGGCATTGAAGTCTCAT	ACAGCCAAGTCTGTGACTTGCACGGTCAT	HEX	Iowa Black FQ	ACAGTCTGTGACTTGCACGGTCAT	6-FAM	Iowa Black FQ	53	93
24	TP53	c.273G>A	CACCAGCAGCTCCTACAC	GCTGCCCTGGTAGGTTT	TCCTGGCCCCTGTCATCTTCTGT	HEX	Iowa Black FQ	TCCTGACCCCTGTCATCTTCTGT	6-FAM	Iowa Black FQ	53	92
25	TP53	c.376-1G>T	TGACTTTCAACTCTGTCTCCTTCCT	CAGTTGGCAAAACATCTTGTTGAG	TTCCTACAGTACTCCC	HEX	Iowa Black FQ	TTCCTACATTACTCCCCT	6-FAM	Iowa Black FQ	52	70
26	TP53	c.375+1G>-	TCTTGCATTCTGGGACAGC	AGAAATGCAGGGGGATACG	AGTCTGTGACTTGCACGGT	HEX	Iowa Black FQ	AGTCTGTGACTTGCAGGTC	6-FAM	Iowa Black FQ	52	104
27	TP53	c.372C>A	GCATTCTGGGACAGCCAAGT	CAGGCATTGAAGTCTCATGGAA	TGACTTGCACGGTCAGT	HEX	Iowa Black FQ	TGACTTGAACGGTCA	6-FAM	Iowa Black FQ	53	78
28	TP53	c.1024C>T	GGCGTGAGCGCTTCGA	GCCTGGGCATCCTTGAGTT	ATGTTCCGAGAGCTG	HEX	Iowa Black FQ	ATGTTCTGAGAGCTGAA	6-FAM	Iowa Black FQ	52	63
28	ERBB2	c.734C>T	ACTGACTGCTGCCATGAGCA	CACATTGGGCACAAAGCAG	TGCACGGGCCCCAA	HEX	Iowa Black FQ	TGCATGGGCCCCAAG	6-FAM	Iowa Black FQ	53	92
31	MET	c.3938A>T	CTCTGGGAGCTGATGACAAG	TGGGCAGTATTCGGGTTGTA	ACTGTTTACTTGTTGCAAGGG	HEX	Iowa Black FQ	ACTGTTTTCTTGTTGCAAGGG	6-FAM	Iowa Black FQ	52	111
31	BRCA1	c.5282delT	CACCAAGGTCCAAAGCGAG	GATCTGTGGGCATGTTGGTG	CAGAAAGATCTTCAGGGGGCT	HEX	Iowa Black FQ	CAGAAAGATCTCAGGGGGCT	6-FAM	Iowa Black FQ	52	100
32	TP53	c.394A>C	TGTCTCCTTCCTCTTCCTACAGTACTC	GCTGCACAGGGCAGGTCTT	CCCTCAACAAGATG	HEX	Iowa Black FQ	TGCCCTCAACCAGA	6-FAM	Iowa Black FQ	52	80
34	TP53	c.839G>A	GCTTTGAGGTGCGTGTTTGTG	TGCGGAGATTCTCTTCCTCTGT	CCTGGGAGAGACCG	VIC	NFQMGB	CCTGGGAAAGACCG	6-FAM	NFQMGB	60	66
35	TP53	c.742C>T	AACTACATGTGTAACAGTTCCTGCAT	GAGTCTTCCAGTGTGATGATGGT	TGGGCCTCCGGTTCA	VIC	NFQMGB	TGGGCCTCCAGTTCA	6-FAM	NFQMGB	52	77
36	TP53	c.527G>A	AGCACATGACGGAGGTTGT	TGCTCACCATCGCTATCTGA	AGGCGCTGCCCCCAC	HEX	Iowa Black FQ	AGGCGCTACCCCCAC	6-FAM	Iowa Black FQ	52	66
