# Supplementary material for: Homologous recombination DNA repair deficiency and PARP inhibition activity in primary triple negative breast cancer
Source: Nat Commun. 2020 May 29;11:2662. doi: 10.1038/s41467-020-16142-7 (PMC7260192; doi:10.1038/s41467-020-16142-7)
Supplement: Supplementary file 2 — Reporting Summary [file 41467_2020_16142_MOESM2_ESM.pdf]

nature research

Corresponding author(s): Prof Nicholas Turner
Last updated by author(s): Feb 12, 2020

Reporting Summary

Nature Research wishes to improve the reproducibility of the work that we publish. This form provides structure for consistency and transparency in reporting. For further information on Nature Research policies, seeAuthors & Referees and theEditorial Policy Checklist .

Statistics

For all statistical analyses, confirm that the following items are present in the figure legend, table legend, main text, or Methods section.

n/a	Confirmed	
☐	☒	The exact sample size (*n*) for each experimental group/condition, given as a discrete number and unit of measurement
☐	☒	A statement on whether measurements were taken from distinct samples or whether the same sample was measured repeatedly
☐	☒	The statistical test(s) used AND whether they are one- or two-sided<br>*Only common tests should be described solely by name; describe more complex techniques in the Methods section.*
☐	☒	A description of all covariates tested
☐	☒	A description of any assumptions or corrections, such as tests of normality and adjustment for multiple comparisons
☐	☒	A full description of the statistical parameters including central tendency (e.g. means) or other basic estimates (e.g. regression coefficient) AND variation (e.g. standard deviation) or associated estimates of uncertainty (e.g. confidence intervals)
☐	☒	For null hypothesis testing, the test statistic (e.g. *F*, *t*, *r*) with confidence intervals, effect sizes, degrees of freedom and *P* value noted<br>*Give P values as exact values whenever suitable.*
☒	☐	For Bayesian analysis, information on the choice of priors and Markov chain Monte Carlo settings
☒	☐	For hierarchical and complex designs, identification of the appropriate level for tests and full reporting of outcomes
☒	☐	Estimates of effect sizes (e.g. Cohen's *d*, Pearson's *r*), indicating how they were calculated

Our web collection on statistics for biologists contains articles on many of the points above.

Software and code

Policy information about availability of computer code

Data collection	Digital PCR data was collected using Quantasoft v1.7 available from BIORAD.<br>Whole genome sequencing was collected using HiSeq Control Software (v3.5.0).<br>Targetted sequencing of tumours was collected using MiSeq Reporter (MSR v2.5.1).<br>AVENIO sequencing of ctDNA was collected using NextSeq system suite (v2.2.0).<br>Bisulfite Sequencing was collected using Miniseq system suite v1.1<br>RNA sequencing was collected using HiSeq Control Software (v2.2.68).
Data analysis	All code used for the analysis of sequencing data is detailed in the specific method sections, with links and is referenced where appropriate.<br>Whole genome sequencing: CaVEMan (Cancer Variants Through Expectation Maximization: http://cancerit.github.io/CaVEMan/), Pindel version 2.0. (http://cancerit.github.io/cgpPindel/), BRASS (BReakpoint AnalySiS) (https://github.com/cancerit/BRASS), and ASCAT (v2.1.1). Annotation was to Ensembl build 75.<br>Targetted tumour sequencing: available by request at https://git.icr.ac.uk/<br>AVENIO ctDNA sequencing: AVENIO Oncology analysis software (v1.0.0 and v1.1) available from Roche.<br>Bisulfite sequencing: Eukaryotic Promoter Database (http://epd.vital-it.ch/index.php, trim-galore (https://www.bioinformatics.babraham.ac.uk/projects/trim_galore/), Biostrings R package.<br>RNA sequencing: STAR aligner (https://github.com/alexdobin/STAR), htseq (https://github.com/simon-anders/htseq), DeSeq2 (DOI: 10.18129/B9.bioc.DESeq2), fgsea (DOI: 10.18129/B9.bioc.fgsea), AIMS (DOI: 10.18129/B9.bioc.AIMS), TNBCtype (http://cbc.mc.vanderbilt.edu/tnbc/) and Cibersort (https://cibersort.stanford.edu/index.php).

For manuscripts utilizing custom algorithms or software that are central to the research but not yet described in published literature, software must be made available to editors/reviewers. We strongly encourage code deposition in a community repository (e.g. GitHub). See the Nature Research guidelines for submitting code & software for further information.

Data

Policy information about availability of data

All manuscripts must include a data availability statement. This statement should provide the following information, where applicable:

- Accession codes, unique identifiers, or web links for publicly available datasets
- A list of figures that have associated raw data
- A description of any restrictions on data availability

All sequencing data from tumour samples that support the findings of this study (Figures 1–5 and Supplementary Figures 1, 3 and 4) is deposited in the European Genome-phenome Archive (EGA), reference EGAS00001004190. Other processed data will be made available on request.

Field-specific reporting

Please select the one below that is the best fit for your research. If you are not sure, read the appropriate sections before making your selection.

- [x] Life sciences
- [ ] Behavioural & social sciences
- [ ] Ecological, evolutionary & environmental sciences

For a reference copy of the document with all sections, see nature.com/documents/nr-reporting-summary-flat.pdf

Life sciences study design

All studies must disclose on these points even when the disclosure is negative.

Sample size	The study size was determined using a Simon two stage Minimax design on Ki67 response in patients with sporadic TNBC, with p0=10% and p1=25% Ki67 response rate. With a two-sided alpha 1.6% and 90% power, four Ki67 responders were required in the first 41 assessable patients to proceed to a full 73 patients. The study would declare inefficacy if <4/41 or <14/73 responses were observed. An initial futility assessment was also planned after 20 evaluable patients had completed rucaparib treatment and consideration would be given to stopping the trial if 0 responses were observed. Additionally, 5%, 1.6% and 1.6% two-sided alphas were allocated to assess rucaparib activity within BRCA1 methylated tumours, RAD51 foci formation tumours, and genomic classifier HRDetect tumours respectively, for a total study two sided alpha of 10%.
Data exclusions	Only patients that completed >7 days of rucaparib treatment were considered for analysis of response.
Replication	IHC results were repeated if assays failed assessed against pre-determined criteria. Analysis was performed by 2 independent scores blinded to each other, time point and clinical details.<br>Sequencing analyses were not replicated, but identification of key mutations validated using dPCR.
Randomization	N/A
Blinding	N/A

Reporting for specific materials, systems and methods

We require information from authors about some types of materials, experimental systems and methods used in many studies. Here, indicate whether each material, system or method listed is relevant to your study. If you are not sure if a list item applies to your research, read the appropriate section before selecting a response.

Materials & experimental systems

n/a	Involved in the study	
[ ]	[x]	Antibodies
[x]	[ ]	Eukaryotic cell lines
[x]	[ ]	Palaeontology
[x]	[ ]	Animals and other organisms
[ ]	[x]	Human research participants
[ ]	[x]	Clinical data

Methods

n/a	Involved in the study	
[x]	[ ]	ChIP-seq
[x]	[ ]	Flow cytometry
[x]	[ ]	MRI-based neuroimaging

Antibodies

Antibodies used	mouse monoclonal anti-Ki67 antibody MIB-1 clone, Dako, M7240.<br>rabbit monoclonal anti-cPARP antibody (Asp214, clone D64E10, Cell Signaling Technology, #5625).<br>RAD51 primary antibody (mouse monoclonal, Genetex, GTX70230)<br>Geminin (GMNN) antibody (rabbit polyclonal, Proteintech 10802-1-AP)

Validation	The laboratory that performed the Ki67 and cPARP immunohistochemistry uses an external quality assurance scheme, UK NEQAS (https://ukneqasiccish.org/modules/). The Ki67 MIB-1 clone is extensively validated and is used internationally for routine clinical work. The cPARP IHC protocol was validated internally.<br>Validation of the dual Rad51/GMNN IHC protocol is presented in Supplementary Figure 2.

Human research participants

Policy information about studies involving human research participants

Population characteristics	The patients enrolled in the RIO study were had a diagnosis of primary triple negative or BRCA1/2 related breast cancer
Recruitment	Key eligibility criteria included breast tumour size ≥2cm or <2cm with cytologically/histologically confirmed axillary lymph nodes, WHO performance status 0-2, no prior history of ipsilateral breast cancer within 5 years and no prior treatment with PARP inhibitors.
Ethics oversight	The RIO trial was given by the NRES Committee London - Fulham Research Ethics Committee (REC ID: 14/LO/2181)

Note that full information on the approval of the study protocol must also be provided in the manuscript.

Clinical data

Policy information about clinical studies

All manuscripts should comply with the ICMJEguidelines for publication of clinical research and a completedCONSORT checklist must be included with all submissions.

Clinical trial registration	EudraCT 2014-003319-12, Cancer Research UK trial CRUK/12/034
Study protocol	ICR Clinical Trials & Statistics Unit (ICR-CTSU),<br>Division of Clinical Studies,<br>The Institute of Cancer Research,<br>Sir Richard Doll Building,<br>Cotswold Road,<br>Sutton, Surrey SM2 5NG.
Data collection	The RIO study was co-sponsored by The Institute of Cancer Research and The Royal Marsden Hospital NHS Foundation Trust. Patients were recruited at six centres across the UK, from August 2015 to December 2017. Central coordination of the study was by the The Institute of Cancer Research Clinical Trials and Statistics Unit (ICR-CTSU).<br>Collection of data for the primary endpoint was performed at the Ralph Lauren Centre for Breast Cancer Research, Royal Marsden Hospital, London from August 2017 to September 2018. Similarly, collection of data for the secondary and exploratory endpoints was performed from August 2017 to September 2018, by the Molecular Oncology Laboratory (Breast Cancer Now Toby Robins Research Centre, ICR, London) together with The Centre for Molecular Pathology (Royal Marsden Hospital, Sutton), The Ralph Lauren Centre for Breast Cancer Research (Royal Marsden Hospital, London), Department of Medical Genetics (The Clinical School, Addenbrooke's Treatment Centre) and MRC Cancer Unit (University of Cambridge, UK).
Outcomes	The primary endpoint was Ki67 response, assessed using immunohistochemistry, from baseline to end of treatment defined as a ≥50% decrease.<br>Secondary and exploratory endpoints included change in circulating tumour DNA levels between baseline and day 12-14 (droplet digital PCR), assessment of a genomic predictor of HR deficiency (whole genome sequencing and HRDetect), BRCA1 methylation (bisulphite sequencing), RAD51 focus deficiency in the end of treatment biopsy (immunohistochemistry), apoptosis by determination of cPARP (immunohistochemistry), and safety and tolerability of rucaparib.
